# From an imbalance to a new imbalance: Italian-style gluten-free diet alters the salivary microbiota and metabolome of African celiac children

**DOI:** 10.1038/srep18571

**Published:** 2015-12-18

**Authors:** Danilo Ercolini, Ruggiero Francavilla, Lucia Vannini, Francesca De Filippis, Teresa Capriati, Raffaella Di Cagno, Giuseppe Iacono, Maria De Angelis, Marco Gobbetti

**Affiliations:** 1 Department of Agricultural Sciences, Division of Microbiology, University of Naples Federico II, Portici, 80055, Italy; 2 Department of Interdisciplinary Medicine, University of Bari Aldo Moro, Bari, 70126, Italy; 3 Department of Agricultural and Food Sciences, University of Bologna, Bologna, 40121, Italy; 4 Inter-departmental Centre for Industrial Agri-Food Research, University of Cesena, Cesena, 47521, Italy; 5 Pediatric Gastroenterology, Di Cristina Children’s Hospital, Palermo, 90134, Italy; 6 Department of Soil, Plant and Food Sciences, University of Bari Aldo Moro, Bari, 70126, Italy

## Abstract

Fourteen Saharawi celiac children following an African-style gluten-free diet for at least two years were subjected to a change of diet to an Italian-style gluten-free diet for 60 days. Significant differences were identified in the salivary microbiota and metabolome when Saharawi celiac children switched from African- to Italian-style dietary habits. An Italian-style gluten-free diet caused increases in the abundance of *Granulicatella*, *Porphyromonas* and *Neisseria* and decreases in *Clostridium, Prevotella* and *Veillonella*, altering the ‘salivary type’ of the individuals. Furthermore, operational taxonomic unit co-occurrence/exclusion patterns indicated that the initial equilibrium of co-occurring microbial species was perturbed by a change in diet: the microbial diversity was reduced, with a few species out-competing the previously established microbiota and becoming dominant. Analysis of predicted metagenomes revealed a remarkable change in the metabolic potential of the microbiota following the diet change, with increased potential for amino acid, vitamin and co-factor metabolism. High concentrations of acetone and 2-butanone during treatment with the Italian-style gluten-free diet suggested metabolic dysfunction in the Saharawi celiac children. The findings of this study support the need for a translational medicine pipeline to examine interactions between food and microbiota when evaluating human development, nutritional needs and the impact and consequences of westernisation.

Celiac disease is an autoimmune disorder that occurs in genetically predisposed individuals (0.8–2.7% of the population)^1^. The highest worldwide prevalence of celiac disease (5.6%) is observed in Saharawi, an African living in the Western Sahara^2^. The frequencies of HLA-DQ2 and DQ8 genotypes within the population are the primary cause^3^. Furthermore, this population changed dietary style during the last century. It moved from the Bedouin diet, based on camel milk and meat, dates, sugar, and small amounts of cereals and legumes, to the high-gluten diet of Saharawi refugees^4^. Celiac disease is particularly severe for Saharawi children, causing chronic diarrhoea, stunting, anaemia, dental anomalies, lactose intolerance, infertility, and nonspecific abdominal pain coupled with multiple deficiency states^4^.

The only safe and effective treatment for celiac patients worldwide is the lifelong exclusion of gluten-containing foods. In Western countries, the gluten-free diet is based on commercial products, which are certified and targeted to celiac patients. In the Saharawi context, the gluten-free diet is mainly based on foods otherwise available and without certification. Under these conditions, cross-contamination with gluten and/or hidden gluten is very difficult to avoid^2^,^5^. The inadequate food availability and the inevitable low compliance to the gluten-free diet have increased the extent of celiac disease related symptoms and have resulted in a high mortality rate. The framework just described, although contextualised to the Saharawi population, may represent a model system common to several African countries.

Oral microbiota may play a pivotal role in health and disease^6^. Saliva is considered an emerging and effective medium for the diagnosis of health and disease^8^. Oral bacteria are not only responsible for human oral diseases^9^ but they have been also associated to non-oral diseases, such as bacterial endocarditis^10^, heart disease^11^, cancer^12^, pneumonia^13^, atherosclerosis^14^, preterm low birth weight^15^, chronic kidney disease^16^, obesity^17^ and pancreatic cancer^18^. Compared with healthy individuals, both the oral and gastro-intestinal microbiota are different during celiac disease pathogenesis in individuals from Western countries^19^,^20^,^21^,^22^,^23^. To the best of our knowledge, no studies have investigated the salivary microbiota of the Saharawi population, which is exceptionally affected by celiac disease. Knowledge of the changes induced by the diet in Saharawi celiac patients can be used to identify the microbial consortia responsible for oral homeostasis and how they are influenced by the intake of specific nutrients. This knowledge may also help with identifying a diet that may improve the health status when implemented. Based on the above premise, this study investigated the salivary microbiota and metabolome of Saharawi celiac children in response to the change from the traditional African- to the Italian-style gluten-free diet.

## Results

### Subject enrolment and clinical evaluation

Fourteen Saharawi children, with biopsy-confirmed celiac disease, being treated with a gluten-free diet for at least two years and following an African-style diet, were enrolled (Table S1). The percentage of Saharawi celiac children who had borderline/positive values for tissue transglutaminase antibody and anti-endomysial antibodies was high (42.85 and 50%, respectively). The mean values for other clinical parameters were within the normal range. The Saharawi celiac children were treated with an Italian-style gluten-free diet for 60 days. The African-style diet was rich in gluten-free cereals, legumes and vegetables and had high carbohydrate, fibre and non-animal protein contents (Table S2). Depending on age, the number of calories consumed ranged from 672 to 996 kcal/d. The Italian-style diet was a typical Western omnivore diet, including animal proteins, sugars, starch, fat and fibres (Table S2).

Saliva samples were collected and analysed for the microbiota and metabolome of Saharawi celiac children. As estimated by plate count on selective media, Bacteroidetes increased (*P* = 0.0007) in saliva samples of Saharawi celiac children during the intervention with Italian-style gluten-free diet compared with the African-style gluten-free diet (Table S3).

### The Italian-style gluten-free diet induced a decrease in salivary microbial diversity

The microbial composition of salivary samples of Saharawi celiac children was analyzed by pyrosequencing of 16S rRNA gene. The bacterial community richness and alpha diversity measures were determined (Figure S1). The analysis revealed a significant decrease in microbial diversity after 30 days of treatment with an Italian-style gluten-free diet. A re-increasing trend was noted after 60 days, although the diversity values were still significantly lower than those registered before the diet change.

### Diet influenced the composition of the salivary microbiota

**Figure 1:**
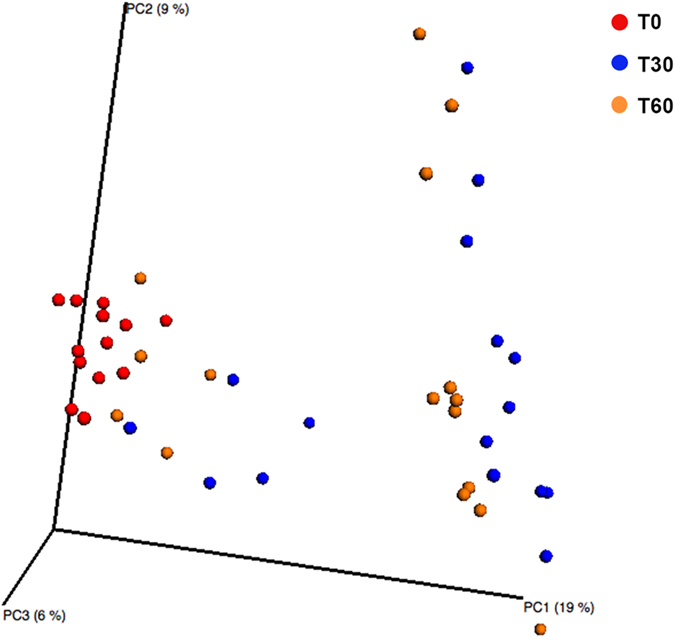
Principle Coordinate Analysis (PCoA). The analysis was based on unweighted UniFrac distance matrix of all 16S rRNA gene sequences found on salivary samples of Saharawi celiac children under African-style gluten-free (T0), and after 30 (T30) and 60 (T60) days of intervention with Italian-style gluten-free diet.

Firmicutes, Actinobacteria and Tenericutes significantly decreased, whereas Proteobacteria increased (*P* = 0.00041) in the saliva of Saharawi celiac children after 60 days on an Italian-style gluten-free diet (Figure S2). No significant (*P* > 0.050) differences were identified for the other phyla. The saliva samples of Saharawi celiac children on an Italian-style gluten-free diet were clearly differentiated as shown by the principal coordinates analysis based on unweighted Unifrac distances (Fig. 1). In addition, both Adonis and Anosim statistical tests based on Unifrac distance matrices indicated a significant influence of diet on the microbial diversity (False Discovery Rate, FDR, < 0.050).

**Figure 2:**
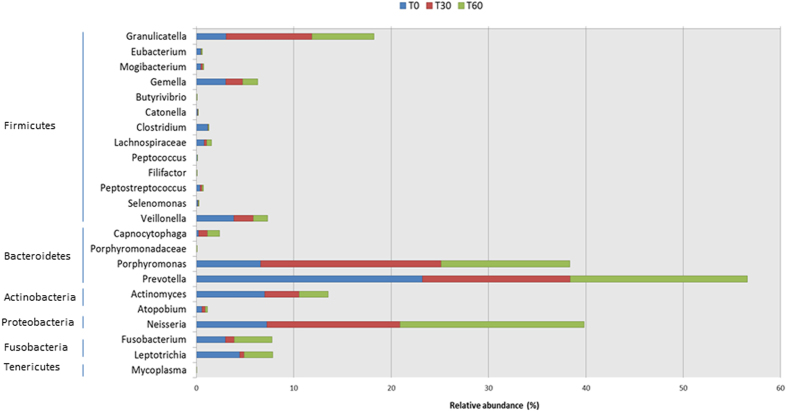
Relative abundance (%) of genera. Relative proportions (%) of predominant bacteria, which showed significant (*P* < 0.050) differences between the salivary samples of Saharawi celiac children under African-style gluten-free diet (T0), and after 30 (T30) and 60 (T60) days of intervention with Italian-style gluten-free diet.

Many significant differences in relative abundance of bacterial genera/species were associated with the diet change (Fig. 2, Figure S3). Within Firmicutes, the abundance of some bacteria belonging to Clostridiales FamilyXIII IncertaeSedis (e.g., *Eubacterium*, *Mogibacterium*), Gemellaceae (*Gemella*), Lachnospiraceae (e.g., *Butyrivibrio*, *Catonella*, *Clostridium*), Peptococcaceae (*Peptococcus*), Peptostreptococcaceae (*Filifactor*, *Peptostreptococcus*) and Veillonellaceae (*Selenomonas*, *Veionella*) significantly decreased during treatment with the Italian-style gluten-free diet compared with the African-style gluten-free diet. By contrast, Carnobacteriaceae (*Granulicatella*) markedly increased under treatment with the Italian-style gluten-free diet. Within the phylum Bacteroidetes, the abundance of Flavobacteriaceae (genus *Capnocytophaga*) and Porphyromonadaceae (*Porphyromonas*) significantly increased with the Italian-style gluten-free diet. An opposite trend was observed for bacteria belonging to Prevotellaceae (*Prevotella*). Compared with the African-style gluten-free diet, the abundance of some bacteria belonging to Burkholderiaceae and Neisseriaceae (*Neisseria*) markedly increased after 60 days of treatment with the Italian-style gluten-free diet.

Operational taxonomic unit (OTU) co-occurrence was investigated by considering the family- (Figure S4) or genus-level (Figure S5) taxonomic assignments and significant correlations at FDR < 0.050. The highest positive correlations were noted between *Actinomycetaceae* and several families belonging to Firmicutes (e.g. Clostridiales family XIII). In addition, the most significant co-exclusion patterns were identified for *Neisseriaceae*/*Neisseria*, *Porphyromonas* and especially *Granulicatella*, which co-excluded many other different genera.

### The salivary microbiome of Saharawi celiac children still differed from those of Italian celiac and healthy children after eating an Italian-style gluten-free diet

**Figure 3:**
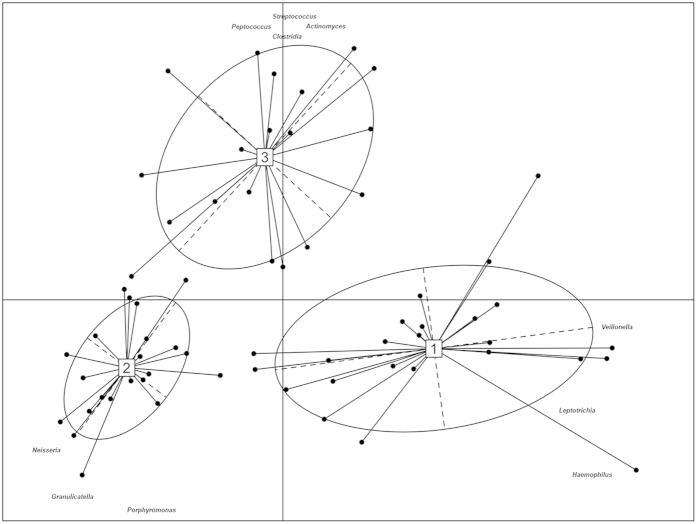
Operational taxonomic unit abundance drives the differentiation of salivary types. Between-class analysis visualizing the results from Principal Component Analysis and clustering based on the Jensen-Shannon distance of the saliva samples analyzed in this study. The center of gravity for each cluster is marked by a rectangle indicating the salivary type, and the ellipse covers 67% of the samples belonging to the cluster. Only operational taxonomic units with loadings >=0.7 in at least one cluster are shown in the figure.

Regardless of the relative abundance, a core salivary microbiome (genus level) was commonly observed in >98% of the Saharawi celiac children both before and after treatment with the Italian-style gluten-free diet (this study), and this core microbiome was similar to those reported in Italian celiac and healthy children in a recent study (Table S4)^23^. This core salivary microbiome consisted of 15 genera belonging to the phyla Firmicutes (*Gemella*, *Granulicatella*, *Mogibacterium*, *Streptococcus* and *Veillonella*), Bacteroidetes (*Porphyromonas*, *Prevotella* and *Capnocytophaga*), Proteobacteria (*Neisseria*, *Haemophilus*), Fusobacteria (*Fusobacterium* and *Leptotrichia*), Actinobacteria (*Actinomyces*) and Tenericutes (*Bulleidia*). The core microbiome of Saharawi celiac children also included other nine genera (*Abiotrophia*, *Atopobium*, *Catonella*, *Clostridium*, *Eubacterium*, *Oribacterium*, *Peptostreptococcus*, *Rothia* and *Selenomonas*). A partitioning around medoid cluster analysis of saliva samples was performed, and the Calinski-Harabasz index indicated that three was the optimal number of clusters (Fig. 3). Supervised clustering methods may force the separation and these clusters are intended as continuous distribution of subjects rather than discrete entities^24^. With this caveat in mind, partitioning around medoids clustering may be still useful to understand which OTUs drive the stratification of the subjects. The abundance of the core genera allowed stratifying the children into three “salivary types”. Cluster I was characterised by a high abundance of *Haemophilus*, *Veillonella* and *Leptotrichia*. Cluster II was distinguished by *Porphyromonas*, *Granulicatella* and *Neisseria* and cluster III by *Streptococcus*, *Actinomyces*, *Peptococcus* and *Clostridia*. The three “salivary types” were significantly different using Adonis (*P* < 0.001) and Anosim (*P* < 0.001). Significant (*P* < 0.050) associations were identified between subject type (Italian healthy children, Italian and Saharawi celiac children) and “salivary type”. Cluster I included only saliva samples from Italian healthy and celiac children^23^. The saliva samples of Saharawi celiac children were grouped into clusters II and III. These two clusters mainly differed based on dietary habits. Cluster III mainly contained saliva samples of Saharawi celiac children treated with an African-style gluten-free diet (85, 29 and 21% of samples taken at 0, 30 and 60 days of diet intervention, respectively). Cluster II contained saliva samples after 30 (71%) and 60 (79%) days of treatment with the Italian-style gluten-free diet. Unweighted Unifrac analysis revealed a clear differentiation between the salivary microbiota of Saharawi and Italian celiac children, which remained sharp even after treatment with an Italian-style gluten-free diet (Figure S6).

### Diet-induced changes in the predicted metabolic functions of the salivary microbiota

**Figure 4:**
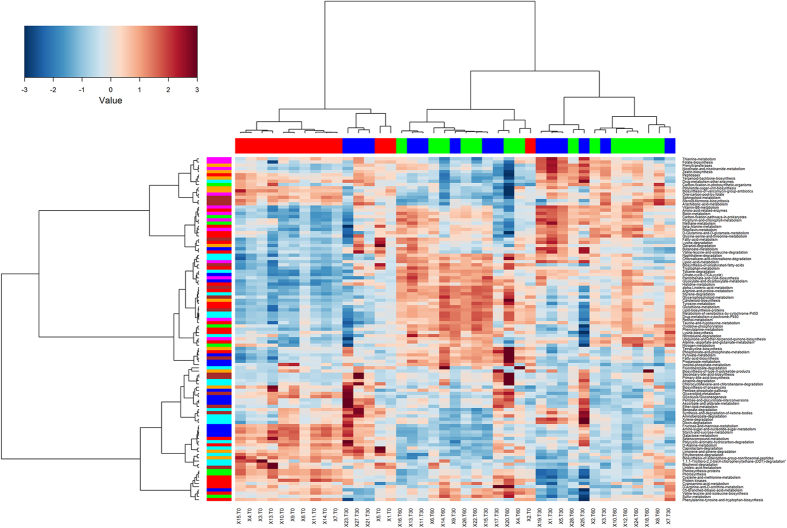
Hierarchical Ward-linkage clustering. The analysis was based on the Spearman correlation coefficients of the proportion of KEGG Orthologs collapsed at level 3 of hierarchy, filtered for subject prevalence of at least 20%. Column bar is color-coded by the time of treatment: subjects under African-style gluten-free diet (t0, red) and after 30 (blue) and 60 (green) days of Italian-style gluten-free diet. Row bar colors denote the higher level of hierarchy (L2): carbohydrate (blue), amino acid (red), energy (green), cofactors and vitamins (magenta), terpenoids and polyketides (orange), lipid (brown) and xenobiotics (cyan) metabolism. Only KEGG Orthologs belonging to metabolism category were considered. The color scale represents the scaled abundance of each gene, denoted as Z-score, with red indicating high abundance and blue indicating low abundance.

Using the phylogenetic investigation of communities by reconstruction of unobserved states, (PICRUSt, http://picrust.github.io/picrust/) as a predictive tool, the potential functions were determined by retrieving the metagenomes. The weighted nearest sequenced taxon index for the samples was 0.074 ± 0.027, indicating a satisfactory level of accuracy for a potential phylogenetic investigation of communities by reconstruction of unobserved states prediction. It was found that human-associated samples have the lowest (best) nearest sequenced taxon index values (0.03 ± 0.20), thanks to the wide number of sequenced genomes available. Other mammalian guts have a higher mean nearest sequenced taxon index value (0.14 ± 0.06), and diverse communities, such as soil, also have much higher nearest sequenced taxon index values (0.17 ± 0.02)^25^. The metagenome prediction confirmed the remarkable effects of the diet change not only on the microbial community composition, but also at a potential functional level. The saliva samples from children on an African diet (T0) could be clearly distinguished from the samples taken after 30 and 60 days of an Italian-style gluten-free diet (Fig. 4). Considering the genes assigned to metabolic functions, the saliva samples at T0 had a higher abundance of genes related to carbohydrate metabolism, whereas after the diet change, the saliva was characterised by a higher abundance of genes related to the metabolism of amino acids, vitamins and cofactors. A procrustes analysis was used to co-visualise the Principal Coordinates Analysis from the 16S-based community structure and predicted metagenomes (Figure S7). In this type of analysis, if the two matrices are perfectly superimposable, the same sample separation can be achieved by using both the datasets. Although the two the Principal Coordinates Analyses were not perfectly superimposable, the procrustes analysis in our case showed that the separation of the samples achieved both at OTU composition and at functional levels was driven by the diet and the length of treatment, with the samples at T0 clustering separately from treated samples.

### Diet influenced the salivary metabolome

**Figure 5:**
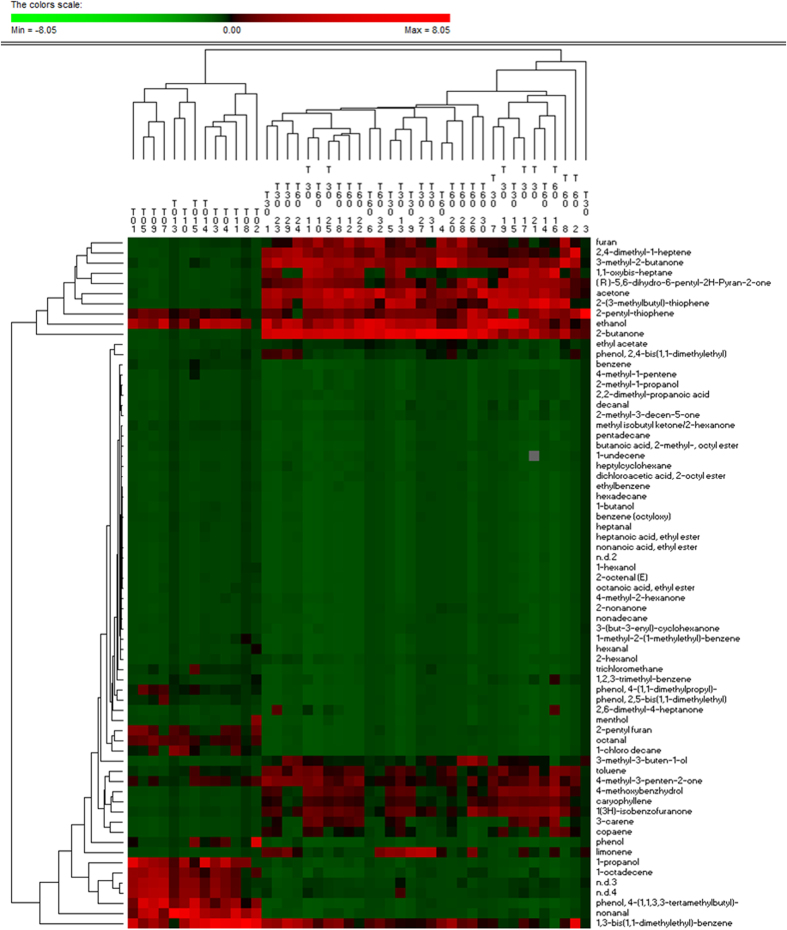
Permutation analysis of volatile organic compounds. Volatile organic compounds were found on salivary samples of Saharawi celiac children under African-style gluten-free diet (T0), and after 30 (T30) and 60 (T60) days of intervention with Italian-style gluten-free diet.

**Figure 6:**
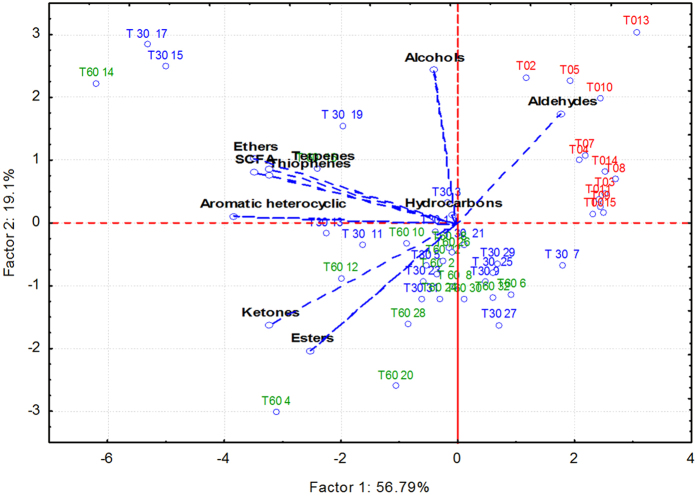
Score plots of the two principal components after Principal Component Analysis of volatile organic metabolites, which were found on salivary samples of Saharawi celiac children under African-style gluten-free diet (T0, red characters), and after 30 (T30, green) and 60 (T60, blue) days of intervention with Italian-style gluten-free diet. All the variables used were listed in Supplementary Table 2. Children were numbered.

Salivary volatile organic compounds were analysed by gas-chromatography mass spectrometry-solid-phase microextraction. Overall, the concentrations of volatile organic compounds increased after 30 and, especially, 60 days of treatment with an Italian-style gluten-free diet (Figure S8). However, the median values for some volatile organic compounds, such as alcohols and phenols (mainly 1-propanol, 4-(1,1,3,3-tetramethylbutyl)-phenol and phenol), aldehydes (heptanal, octanal and nonanal), aromatic heterocyclics (2-pentyl furan) and some hydrocarbons (1,3-bis 1,1-dimethylethyl-benzene; 1-chloro decane; 1-octadecene, benzene and trichloromethane) were significantly (*P* < 0.050) lower in Saharawi celiac children after 30 and, especially, 60 days of treatment with an Italian-style gluten-free diet (Table S5). Ketones (acetone, 2-butanone and 3-methyl-butanone) noticeably increased during treatment with an Italian-style glute-free diet. Permutation and Principal Component Analysis analyses based on gas-chromatography mass spectrometry data clearly distinguished the saliva samples of Saharawi children according to diet (Figs 5 and 6).

### Diet influenced the OTU-metabolite correlations

Overall, the OTU-metabolite correlations changed during treatment with an Italian-style gluten-free diet. For instance, *Actinomyces*, which had the highest abundance in saliva samples of Saharawi celiac children on an African-style gluten-free diet, showed positive correlations (FDR < 0.050) with volatile organic compounds that were found before and after 30 days of an Italian-style gluten-free and negative correlations with other volatile organic compounds detected at 60 days (Figure S9). Genera with the highest abundance during intervention with the Italian-style gluten-free diet showed negative correlations (FDR < 0.050) with some antimicrobial compounds, which were detected at the highest levels in the saliva samples of Saharawi celiac children on an African-style gluten-free diet (e.g., *Porphyromonas* and *Neisseria* versus nonanal) (Figure S9, panel T0). By contrast, genera abundantly associated with an African-style gluten-free diet were negatively correlated with the volatile organic compounds mainly detected under treatment with an Italian-style gluten-free diet (e.g., *Clostridium* versus furan, and *Eubacterium* versus ethyl acetate) (Figure S9, panel T30).

### Correlations between diet, microbiota and metabolome

**Figure 7:**
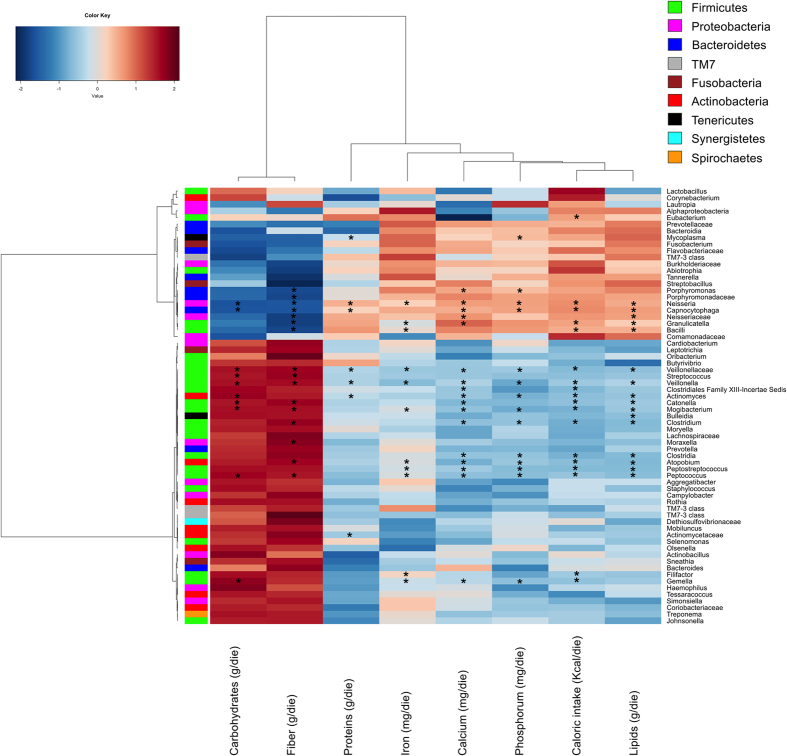
Correlation between dietary information and salivary microbiota composition. Heatplot showing Spearman’s correlations between microbial genera (filtered by a subject prevalence of 5%) and dietary information. Rows and columns are clustered by Euclidean distance and Ward linkage hierarchical clustering. The intensity of the colours represents the degree of association between the genera and the nutrients as measured by the Spearman’s correlations. Row bar is colour-coded by phylum. Asterisk indicates significant correlations after p-value correction (FDR < 0.05).

Plotting the correlation between genera and dietary information, an influence of the diet on the structure of the microbiota could be detected (Fig. 7). Two major clusters of genera and two clusters of nutritional ingredients were detected. Within Bacteroidetes, Porphyromonadaceae (*Porphyromonas*), and *Capnocytophaga* showed negative correlations with carbohydrates and, especially, fiber intake and positive correlations with proteins, iron, calcium, phosphorus, caloric intake and/or lipids. Similar trend was also found for *Neisseria* (Proteobacteria) and *Granulicatella* (Firmicutes). An opposite trend was found for other Firmicutes such as Veillonellaceae (*Veionella*), *Streptococcus*, *Catonella*, *Mogibacterium*, *Clostrium*, *Peptococcus* and *Gemella* and Actinobacteria (*Actinomyces*).

Carbohydrates, fiber and caloric intakes strongly correlated (R > 0.7) with several hydrocarbons (*P* < 0.001) (benzene, tricholoromethane and 1-octadecene), aldehydes (*P* < 0.003) (heptanal, nonanal and octanal) and alcohols (*P* < 0.045) (1-butanol, 1-propanol, and phenol) (Figure S10). Calcium, phosphorum and iron intakes showed the highest positively correlations (R > 0.7) with furans (*P* < 0.031), acetone (*P* < 0.002), ethyl acetate (*P* < 0.002), 2-butanone (*P* < 0.006) and 3-methyl-2-butanone (*P* < 0.009). Furans, acetone, ethyl acetate, 2-butanoneand 3-methyl-2-butanone were also correlated (R > 0.54; *P* < 0.042) with the intake of proteins. Lipids were strongly correlated with acetone (R = 0.764; *P* = 0.001) and toluene (R = 0.617, *P* = 0.032).

## Discussion

Both celiac disease and recurrent enteric infections predispose Western Saharan children to macro- and micro-nutrient deficiencies, impaired intestinal mucosal barrier function, and dental caries and fluorosis^26^,^27^. Other environmental factors (e.g., dysbiosis of the salivary and intestinal microbiota and intestinal infections) may also contribute to the general health status of celiac patients^3^,^20^. In this study, a group of Saharawi celiac children who were staying in Italy as part of a humanitarian project were given a gluten-free diet consistent with Italian dietary habits using certified gluten-free products from the market.

In accordance with the World Health Organisation Global Oral Health Programme, the effect of the Italian-style gluten-free diet on the salivary microbiota was investigated. Currently, saliva is thoroughly studied in both general and dental medicine to search for health and disease biomarkers^28^,^29^,^30^. Saharawi celiac children showed similar alpha-diversity compared with the salivary microbiota of Italian celiac children^23^. Previously, it was hypothesised that lifestyle and diet could affect the salivary microbiota^31^,^32^,^33^. The type of relationship was not fully elucidated. Saliva samples of Saharawi celiac children on an African-style gluten-free diet showed an unusual and remarkable occurrence of *Clostridium* and high abundance of some Firmicutes (*Eubacterium*, *Mogibacterium*, *Catonella*, *Peptococcus*, *Filifactor*, *Peptostreptococcus*), Actinobacteria (*Actinomyces*, *Rothia*) and Tenericutes (*Bulleidia*), which indicated some sort of imbalance in salivary microbiota. The main bacteria involved in gluten degradation at the salivary or intestinal level are Firmicutes (e.g., *Clostridium*) and Actinobacteria (e.g., *Actinomyces, Rothia*)^34^,^35^. The abundance of these OTUs can be attributed to environmental and dietary factors because all of the above genera decreased after the children stayed in Italy and ate an Italian-style gluten-free diet. An Italian-style gluten-free diet led to increases in *Granulicatella*, *Capnocytophaga, Porphyromonas* and *Neisseria*. As shown by partitioning around medoids clustering, the increases in such OTUs led to a significant change in the salivary microbiota, resulting in the identification of a specific salivary type for Saharawi children treated with an Italian-style gluten-free diet. By contrast, *Prevotella* and *Veillonella* decreased after 60 days of treatment with an Italian-style gluten-free diet. Italian-style gluten-free diet triggered the growth of *Porphyromonas*, *Neisseria* and, especially, *Granulicatella* that showed strong exclusion patterns with all of the other OTUs. This study demonstrated that a change in diet can rapidly modify the salivary microbiota and can lead to a change in the “salivary type” of the individuals that are subjected to a different diet. By contrast, diet changes cause significant modifications in the microbiota of the gut, but these modifications do not necessarily cause a change in the enterotype^36^,^37^. In saliva, an initial equilibrium of co-occurring microbial species is perturbed by a change of diet, the microbial diversity is reduced, and a few OTUs become dominant, outcompeting the previously established microbiota. Individual interactions are essential for community stability of the microbiota of all ecosystems. Microbes may exchange or compete for nutrients, signalling molecules and immune evasion mechanisms^38^. The analysis of predicted metagenomes was absolutely consistent with a change in the metabolic potential of the microbiota following the diet change. The Italian-style gluten-free diet had a higher intake of animal proteins, fats, calcium and phosphorus. First, this study revealed a positive associations at salivary level of *Porphyromonas*, *Neisseria* and *Granulicatella* with the intake of proteins, lipids and/or minerals. Consistently, the structure of the microbiota adapted to the Italian-style diet displayed a different metabolic potential where amino acid, vitamin and co-factors metabolisms were apparently increased. A reduction in oral microbial diversity is generally associated with dysbiosis and a predisposition to disease^8^. In addition, the predominant genera of *Granulicatella*, *Neisseria* and *Porphyromonas* found after treatment with the Italian-style gluten-free diet all include species often associated with infection and disease^39^,^40^,^41^,^42^. Therefore, if the celiac children on an African-style gluten-free diet had imbalanced oral microbiota due to their disease state and diet, the westernisation of their diet apparently led to a different type of imbalance, favouring the growth of only a few microbial genera. Understanding how the diet and nutritional status influence the composition and dynamic operations of human microbial communities and the innate and adaptive immune system represents an area of scientific need, opportunity and challenge^27^. Strong positive and negative correlations were identified between the core bacterial genera and volatile organic compounds. Several antimicrobial volatile organic compounds (e.g., 1-propanol, 1-octadecene, octanal, nonanal, phenols, furans, ethyl acetate)^43^,^44^,^45^ were present in the OTU/volatile organic compound co-occurrence/exclusion pattern. In addition, the predicted metabolic pathways based on the identified 16S genes showed the lowest metabolic activity in the salivary microbiota of Saharawi celiac children on an African-style gluten-free. This hypothesis was supported by finding that the lowest volatile organic compound levels were observed with an African-style gluten-free diet. Based on the results of this study, it appears that diet and salivary microbiota affect the balance of the salivary metabolome, which, in turn, alters the colonisation and growth of other bacteria. Compared to Italian celiac and healthy children^23^, Saharawi celiac children subjected to African-style gluten-free diet had a greater presence of volatile organic compounds. The salivary metabolomes of Saharawi celiac children after 30 or 60 days of treatment with an Italian-style gluten-free diet differed markedly from those recently described for Italian celiac and healthy children^23^. In addition, different metabolic responses to the same diet (Italian-style gluten-free diet) between Saharawi and Italian celiac children could be hypothesised. Compared to Italian celiac and healthy children^23^, Saharawi celiac children showed a marked increase in ketones (e.g., acetone and 2-butanone) on an Italian-style gluten-free diet. Acetone, 2-butanone and other ketones were positively associated with the intake of lipids, proteins and minerals. In humans, acetone is the final product of the ketone-body pathway, which supplies the body with a secondary source of energy. The human microbiota affects nutrient processing by the host, including the expression of host genes that are responsible for nutrient transport and metabolism^26^. It was hypothesised that malnutrition affects the metabolic capacity of the gut microbiome^46^. Based on the ketone levels^47^, the Saharawi celiac children seem to show a higher energy recovery from the Italian-style gluten-free diet compared with Italian celiac and healthy children. In agreement with this finding, undernutrition was correlated with being overweight/obese in adolescents living in a rural South African community^48^. Previously, it was reported that undernutrition during the foetal period and/or early childhood leads to metabolic adaptations that may result in obesity later in life^49^.

Overall, the data in this study showed that maintaining the gluten-free diet status but changing the dietary habits (African- to Italian-style) caused the salivary microbiota and metabolome of Saharawi celiac children to undergo significant changes. A few microbial species take over as a result of the diet change and outcompete the previous microbial consortia, thus perturbing the oral microbiota. The high concentrations of some metabolites (e.g., acetone and 2-butanone) during treatment with the Italian-style gluten-free diet suggested metabolic dysfunctions in Saharawi celiac children. Despite living in Italy, adopting the same Italian-style gluten-free diet and adopting the same hygiene conditions, Saharawi celiac children showed different salivary microbiota and metabolomes than Italian celiac children. The findings of this study support the need for a translational medicine pipeline to examine interactions between food and microbiota when evaluating human development, nutritional needs, physiological variations and the impact of westernisation.

## Methods

### African cohort

Fifty Saharawi children with celiac disease living in the refugee camps who were receiving treatment with a gluten-free diet for at least two years (index cases) were invited to participate in this study with informed consent. The children were initially diagnosed by the authors and were treated during previous celiac disease screening programmes. Demographic and clinical data were recorded with the help of Arab-speaking health personnel. All fifty Saharawi celiac children were moved to “G. Di Cristina” Children’s Hospital in Palermo, Italy. They received a check-up, and their health conditions were determined. At this time, prior to the dietary change, saliva and blood samples were taken from all participants for further analysis. Children were then moved to the Institute “A. Salamone” in Palermo, Italy and were treated for approximately seventy days with an Italian-style gluten-free diet.

### Experimental design

This study was approved by the Institutional Review Board of the “G. Di Cristina” Children’s Hospital, and informed written consent was obtained from parents. All experiments were performed in accordance with relevant guidelines and regulations. The exclusion criteria were as follows: presence of neurological or gastrointestinal disease; immunological or autoimmune disorder; a plaque index greater than one; caries; and enamel defects. Subjects included in the study were not treated with antibiotics and/or functional foods (probiotics and/or prebiotics). Samples were collected at the first visit (see above) (T0) and after 30 (T30) and 60 days (T60) of an Italian-style GFD. Fourteen African children (median age 8.4 ± 0.7 years) completed the study. The information and characteristics of the recruited children are reported in Table S1. Blood samples were analysed as described by Teresi *et al.* (2010)^2^.

### Collection of saliva samples

Unstimulated whole saliva samples were collected in the morning, 2 hours after tooth brushing, by direct saliva release into a sterile plastic tube. No intake of food or drink was allowed in the morning before sampling. The samples were either immediately subjected to analysis (for plate counts) or frozen at –20 °C (for DNA extraction and metabolome analyses). For viable counts, saliva samples (1 g) were mixed with 9 ml sterilised physiological solution and homogenised. Counts of viable bacterial cells were determined as described by Francavilla *et al.* (2014)^23^ and outlined in the Supplementary Materials and Methods.

### 16S rRNA gene amplicon library preparation and sequencing

Microbial DNA extraction was performed using the Biostic^TM^ Bacteremia DNA isolation kit (MoBIO Laboratories, Inc. Carlsbad, CA, USA) with 2 ml of saliva sample. The microbial diversity was assessed by pyrosequencing of the amplified (520 bp) V1-V3 region of the 16S rRNA gene using a 454 GS Junior platform (454 Life Sciences, Roche Diagnostics, Italy). Library preparation and sequencing were performed as previously described^50^.

### Bioinformatics, data analysis and metagenome prediction

Raw reads were first filtered according to the 454 processing pipeline. The sequences were then denoised, further filtered and analysed using QIIME 1.8.0 software, using the same pre-processing pipeline recently described^33^. Briefly, the reads were excluded from the analysis if they had an average quality score lower than 25, if they were shorter than 300 bp and if there were ambiguous base calls or primer mismatches. OTUs were picked at 97% of similarity using the open-referenced uclust^51^ method and representative sequences of each cluster were submitted to the RDPII classifier^52^ to obtain the taxonomy assignment by using Greengenes 16S rRNA gene database (version 05/2013)^53^. Alpha and beta diversity were calculated in QIIME, as reported by De Filippis *et al.* (2013)^54^. Clustering analysis was performed by adding raw sequence data from the salivary microbiota of Italian celiac children, which was collected in a previous study^23^. Samples were clustered using the Jensen-Shannon distance and partitioning around medoids clustering, as recently described^32^. Weighted and unweighted UniFrac distance matrices and OTU tables were used to perform ADONIS and ANOSIM statistical tests through the compare_category.py script of QIIME to verify the influence of the time of treatment on the microbial population. Further details about bioinformatics and data analysis are outlined in the Supplementary Materials.

The 16S rRNA gene sequences are available in the Sequence Read Archive of NCBI (accession number SRP247490). PICRUSt, a bioinformatics tool that predicts the abundance of a gene family based on the 16S-based structure of the microbiota^25^, was used to investigate the functional profiles in the salivary microbiota (Supplementary Materials).

### Gas-chromatography mass spectrometry-solid-phase microextraction analysis of salivary volatile compounds

The analyses were performed using a carboxen/polydimethylsiloxane (85 μm) and a manual solid phase micro-extraction holder (Supelco Inc., Bellefonte, PA, USA). Gas-chromatography mass spectrometry analyses were performed with an Agilent 7890A gas chromatograph (Agilent Technologies, Palo Alto, CA, USA) coupled to an Agilent 5975C mass selective detector operating in electron impact mode (ionisation voltage, 70 eV). A Varian CP7773 Wax 52 CB capillary column (length, 50 m; inside diameter, 0.32 mm; Agilent Technologies) was used. The analysis was performed as described by Francavilla *et al.* (2014)^23^ and outlined in the Supplementary Materials and Methods.

### Statistical analyses

Culture-dependent and metabolomics data were obtained in at least triplicate. The analysis of variance (ANOVA) was performed on transformed data followed by separation of means with Tukey’s honest significant difference test using the statistical software Statistica for Windows (Statistica 6.0 per Windows 1998, StatSoft). Differences were considered to be significant at *P* < 0.05 or *P* < 0.01. Permutation analysis was also performed for the metabolomics data. The correlation analysis was conducted using the psych package in the R environment to identify patterns of co-occurrence/exclusion between OTUs or between OTUs and metabolites. Multiple-testing of corrected pairwise Spearman correlations was computed between OTUs at the genus level (abundance > 0.1% in at least 5 samples) and metabolites or between OTUs at the genus and family levels. Co-occurrence/exclusion matrices were plotted using the corrplot package in R. Only significant correlations (FDR < 0.050) were considered.

## Additional Information

**How to cite this article**: Ercolini, D. *et al.* From an imbalance to a new imbalance: Italian-style gluten-free diet alters the salivary microbiota and metabolome of African celiac children. *Sci. Rep.* **5**, 18571; doi: 10.1038/srep18571 (2015).

## Supplementary Material

Supplementary Information
