# Supplementary material for: From an imbalance to a new imbalance: Italian-style gluten-free diet alters the salivary microbiota and metabolome of African celiac children
Source: Sci Rep. 2015 Dec 18;5:18571. doi: 10.1038/srep18571 (PMC4683525; doi:10.1038/srep18571)

Supplementary Information

From an imbalance to a new imbalance: Italian-style gluten-free diet alters the salivary microbiota and metabolome of African celiac children

Danilo Ercolini[1+], Ruggiero Francavilla[2+], Lucia Vannini[3,4], Francesca De Filippis[1], Teresa Capriati[5], Raffaella Di Cagno[6], Giuseppe Iacono[5], Maria De Angelis[6*], Marco Gobbetti[6]

[1]Department of Agricultural Sciences, Division of Microbiology, University of Naples Federico II, Portici, 80055, Italy;

[2]Department of Interdisciplinary Medicine, University of Bari Aldo Moro, Bari, 70126, Italy;

[3]Department of Agricultural and Food Sciences, University of Bologna, Bologna, 40121, Italy;

[4]Inter-departmental Centre for Industrial Agri-Food Research, University of Cesena, Cesena, 47521, Italy;

[5]Pediatric Gastroenterology, Di Cristina Children's Hospital, Palermo, 90134, Italy;

[6]Department of Soil, Plant and Food Sciences, University of Bari Aldo Moro, Bari, 70126, Italy

E-mail address: maria.deangelis@uniba.it

Supplementary Materials and Methods

Enumeration of cultivable bacteria. The following selective media were used: Plate count agar (total facultative aerobes and anaerobes); de Man, Rogosa and Sharpe agar (lactobacilli and enterococci); *Bifidobacterium* agar modified (bifidobacteria) (Becton Dickinson France SA, Le Pont de Claix, France); Glucose-M17 (lactococci and streptococci); Baird Parker plus Egg Yolk Tellurite Emulsion (stafilococci and micrococci); Wilkins-Chalgren anaerobe agar (total anaerobes); Wilkins-Chalgren anaerobe agar plus GN selective supplements and sheep blood defibrinated (*Bacteroides*, *Porphyromonas* and *Prevotella*); Violet red bile agar (Enterobacteria); and Slanetz and Bartley (Enterococci). Except for *Bifidobacterium* agar modified all media were purchased by Oxoid Ltd (Hampshire, England).

Bioinformatics, data analysis and metagenome prediction. Raw reads were first filtered according to the 454 processing pipeline. The sequences were then analyzed and further filtered using QIIME 1.8.0 software.[1] In order to guarantee a higher level of accuracy in terms of Operational Taxonomic Units detection, after the split library script performed by QIIME, the reads were excluded from the analysis if they had an average quality score lower than 25, if they were shorter than 300 bp and if there were ambiguous base calls. After filtering and denoising[2], Operational Taxonomic Units (OTUs) defined by a 97% of similarity were picked using the uclust method[3] and the representative sequences were submitted to the RDPII classifier[4] to obtain the taxonomy assignment using the Greengenes 16S rRNA gene database[5]. Alpha and beta diversity were carried out in QIIME, as reported elsewhere[6]. The correlation analysis was carried out using the psych package in R environment to identify patterns of co-occurrence/exclusion between OTUs or between OTUs and metabolites. Multiple-testing corrected pairwise Spearman correlations were computed between OTUs at the genus level (abundance >0.1% in at least 5 samples) and metabolites or between OTUs at genus and family level. Co-occurrence/exclusion matrices were

plotted using the corrplot package in R. Only significant correlations (False Discovery Rate, FDR, <0.05) were considered.

Clustering analysis was carried out adding sequence data of salivary microbiota from Italian celiac children from a previous study[7]. Samples were clustered using the Jensen–Shannon distance and partitioning around medoids clustering; the optimal number of clusters was estimated using the Calinski–Harabasz index, and the silhouette validation technique was used for assessing the robustness of clusters, as previously described[8]. The analysis was carried out in the R environment using the cluster and ade4 packages. Weighted and unweighted UniFrac distance matrices and OTUs tables were used to perform ADONIS and ANOSIM statistical tests through the compare category.py script of QIIME to verify the influence of the time of treatment on the microbial population.

Pyrosequencing analysis yielded an average of 5898 reads (average length 473 bp) per sample after the quality filters and the estimated sample coverage was above 95% for all the samples.

For analysis with PICRUSt, the OTUs at 97% identity were closed reference picked against the Greengenes database (version 05/2013) using QIIME 1.8. Data were normalised for 16S copy numbers, and the metagenomes were predicted. From the inferred metagenomes, Kyoto Encyclopedia of Genes and Genomes (KEGG) orthologues were identified, and the table obtained was rarefied at the lowest number of sequences/samples. KEGG orthologues were then collapsed at level 3 of the hierarchy, and the resulting table was imported in R (www.r-project.org). The made4 package was used to produce a heatplot using Hierarchical Ward-linkage clustering based on the Spearman correlation coefficients of the proportion of activities belonging to metabolism pathways, filtered for prevalence in at least 20% of the subjects. NSTI were calculated to evaluate the accuracy of metagenome predictions, which depend on how closely related the microbes in a given sample are to microbes with sequenced genome representatives; a lower NSTI value indicates a closer mean relationship [9]. To compare the separation achieved for the samples considering both of the datasets (microbiota and the predicted metagenomes) non-phylogenetic Principal Coordinates

Analysis (PCoA) was performed in QIIME, and the two PCoAs were then combined in a Procrustes analysis.

Gas-chromatography mass spectrometry-solid-phase microextraction analysis of salivary volatile compounds. After preconditioning, according to the manufacturer's instructions, a carboxen/polydimethylsiloxane (CAR/PDMS) (85 µm) and a manual solid phase micro-extraction holder (Supelco Inc., Bellefonte, PA, USA) were used. Before headspace sampling, the fiber was exposed to GC inlet for 5 min for thermal desorption at 250°C. Three grams of salivary sample were placed into 10 mL glass vials and added of 10 µL of 4-methyl-2-pentanol (final concentration of 33 mg/L), as the internal standard. Samples were then equilibrated for 10 min at 45°C. Solid phase micro-extraction fiber was exposed to each sample for 40 min. Both equilibration and absorption phases were carried out with stirring. The fiber was then inserted into the injection port of the gas chromatograph for 10 min of sample desorption. Gas-chromatography mass spectrometry analyses were carried out with an Agilent 7890A gas chromatograph (Agilent Technologies, Palo Alto, CA) coupled to an Agilent 5975C mass selective detector operating in an electron impact mode (ionization voltage, 70 eV). A Varian CP7773 Wax 52 CB capillary column (length, 50 m; inside diameter, 0.32 mm; Agilent Technologies) was used. The temperature program was 40°C for 1 min, followed by an increase, at a rate of 4.5°C/min, to 65°C, an increase, at a rate of 10°C/min, to 230°C, and then 230°C for 17 min. The injector, interface and ion source temperatures were 250, 250, and 230°C, respectively. The mass-to-charge ratio interval was 30 to 350 Da at a rate of 2.9 scans per s. Injection was carried out in splitless mode, and helium (flow rate, 1 mL/min) was used as the carrier gas. Molecules were identified based on comparison of their retention times with those of pure compounds (Sigma-Aldrich, Milan, Italy). Identities were confirmed by searching mass spectra in the available databases (NIST, version 2005; Wiley, version 1996). All the gas-chromatography mass spectrometry raw files were converted to netCDF format via Chemstation (Agilent Technologies) and subsequently processed with the XCMS toolbox

(http://metlin.scripps.edu/download/). XCMS software allows automatic and simultaneous retention time alignment, matched filtration, peak detection and peak matching. The resulting table containing information such as peak index (retention time-m/z pair) and normalized peak area was exported into R (www.r-project.org) for subsequent statistical or multivariate analyses. Quantitative data for the compounds identified were obtained by the interpolation of the relative areas versus the internal standard area.

References

1 Caporaso, J. G. *et al.* QIIME allows analysis of high-throughput community sequencing data. *Nat Methods* **7**, 335–336 (2010).

2 Reeder, J. & Knight, R. Rapidly denoising pyrosequencing amplicon reads by exploiting rank-abundance distributions. *Nat. Methods* **7**, 668–669 (2010).

3 Edgar, R. C. Search and clustering orders of magnitude faster than BLAST. *Bioinformatics* 26, 2460–2461(2010).

4 Wang, Q. *et al*. Naı̈ve Bayesan classifier for rapid assignment of rRNA sequences into the new bacterial taxonomy. *Appl. Environ. Microbiol.* **73**, 5261–5267 (2007).

5 McDonald, D. *et al.* An improved Greengenes taxonomy with explicit ranks for ecological and evolutionary analyses of bacteria and archea. *I.S.M.E. J.* **6**, 610–618 (2012).

6 De Filippis, F. *et al*. Exploring the sources of bacterial spoilers in beefsteaks by culture-independent high-throughput sequencing. *PLoS One* **8**, e70222 (2013).

7 Francavilla, R. *et al*. Salivary microbiota and metabolome associated with celiac desease. *Appl. Environ. Microbiol.* **80**, 3416–3425 (2014).

8 Arumugam, M. *et al*. Enterotypes of the human gut microbiome. *Nature* **473**, 174–180 (2011).

9 Langille, M. G. I. *et al.* Predictive functional profiling of microbial communities using 16S rRNA marker gene sequences. *Nat. Biotechnol.* **31**, 814–821 (2013).

Supplementary Table 1. Baseline demographic and clinical characteristics of Saharawi celiac children who completed the study.

	**Celiac children (n = 14)<br>Baseline**	**Celiac children (n = 14)<br>End of Follow-up**	**P value**
Mean age ± SD (years)	8.4±0.7	8.4±0.7	-
Male/female	4/10	4/10	-
Feeding habits	Gluten-free diet under Africa-style dietary habits	Gluten-free diet under Italian-style dietary habits	-
Calories (Kcal/d)	672.2 – 996.0	1,068.0 - 1,512.0	0.018
BMI centile	14.7±1.0	15.8 ± 1.1	0.042
Tooth brushing habits	2 times a day	2 times a day	-
Glycaemia mg/dL)	80.93±5.03	81.50±5.03	0.157
Na (mmol/L)	137.5±1.09	137.18±1.33	0.276
K (mmol/L)	3.87±0.20	3.89±0.22	0.840
Cl (mmol/L)	104.0 ±1.17	104.0 ±1.09	0.961
Prot tot (g/dL)	6.83±0.39	6.88±0.39	0.351
Col tot (mg/dL)	145.7±18.51	145.8±17.26	0.128
HDL (mg/dL	37.3±7.20	37.2±7.00	0.351
Triglycerides (mg/dL)	54.1±9.99	54.8±10.80	0.084
Hemoglobin (g/Dl)	12.1±1.95	12.3±1.86	0.123
PLT (cell/µL)	349,357±88.656	339,125±87.143	0.314
AST (U/L)	36.5±5.47	34.75±7.12	0.099
GGT (U/L)	14.8±3.42	15.7±4.95	0.128
BIL tot (mg/dL)	0.4±0.10	0.4±0.14	0.934
Ab antitTG IgA (U/mL)	8 children <10; 6>10	8 children <10; 6>10	0.152
Ab antiTG (UI/mL)	<20	<20	0.437
Ab antitTG IgG(U/mL)	10 children <6; 4>6	10 children <6; 4>6	0.182
EMA IgA	7 children -; 7 +	7 children -; 7 +	0.140
TSH (microUI/mL)	2.4±1.59	2.1±1.61	0.541
FT3 (pmol/L)	8.4±1.00	8.2±1.02	0.283
FT4 (ng/dL)	1.1±0.11	1.1±0.12	0.098
Ab antiTPO (UI/mL)	14.9±8.68	14.3±7.09	0.522
Ferritin (ng/mL)	25.36±24.5	23.19 ± 23.3	0.774

Supplementary Table 2. Common foods ingested by Saharawi celiac children during the 24 hours before being examined and dietetic daily intake (median values) of macro and micro nutrients of African and Italian children.

Food	African cumulative frequency of food	Italian cumulative frequency of food	P value
Bread/pasta	88	100	0.048
Rice	70	45	0.002
Potatoes	30	40	0.087
Meat	20	50	0.012
Milk	20	80	0.041
Couscous	20	0	0.000
Cheese	15	70	0.002
Oil	15	100	0.001
Lentils	15	30	0.017
Dates	7	0	0.001
Eggs	5	30	0.003
Fish	3	25	0.010
**Dietetic Composition**	**Saharawi**	**Italy**	
Caloric Intake/die (Kcal)	834	1947	0.041
Carbohydrate Intake /die (g)	153	120	0.012
Lipid Intake /die (g)	25.4	34	0.044
Proteic Intake /die (g)	31.3	35.5	0.047
Daily Calcium Intake (mg)	200	1000	0.003
Daily Phosphorum Intake (mg)	480	1000	0.008
Daily Iron Intake (mg)	3.9	9	0.012
Daily Fiber Intake (g)	21	11	0.009

Supplementary Table 3. Median values and range of cultivable cells (log CFU/mL) of the main microbial groups of salivary samples of Saharawi celiac children under African-style gluten-free diet (T0), and after 30 (T30) and 60 (T60) days of intervention with Italian-style gluten-free diet.

**Microbial group**	**T0**	**T30**	**T60**	**P value**		
				**T0 *vs* T30**	**T0 *vs* T60**	**T30 *vs* T60**
Total aerobic bacteria	5.00 (4.51-6.00)	5.36(2.70-6.08)	5.41(3.19-6.47)	0.678	0.826	0.846
Total anaerobes	6.00 (4.51-7.00)	6.21(4.11-7.18)	6.26(4.00-7.34)	0.988	0.922	0.712
Staphylococcus and *Micrococcus*	2.48 (1.00-4.63)	1.00 (1.00-3.79)	1.00 (1.00-3.85)	0.722	0.732	0.423
Bacteroides, Porphyromonas and *Prevotella*	2.18 (2.00-5.04)	3.19(3.00-6.08)	4.00(3.00-6.51)	0.000	0.027	0.219
Enterobacteriaceae	1.00 (0.00-1.2)	1.00(0.00-2.00)	1.04(0.00-1.50)	0.423	0.351	0.471
Enterococcus and Lactobacillus	5.41(4.26-6.18)	5.22(1.00-6.49)	5.87(1.00-6.45)	0.447	0.558	0.459
Lactococcus and Streptococcus	4.90(4.23-5.60)	4.90(1.00-6.46)	4.87(2.95-6.14)	0.591	0.680	0.533
Bifidobacterium	5.00(1.00-6.51)	5.22(1.00-7.00)	5.16(1.00-6.03)	0.950	0.968	0.913

Data are the means of three independent experiments (n = 3) for each subject. Statistics: t-test; the alpha level: two-tailed.

Supplementary Table 4. Relative proportion of predominant bacterial taxa, which were found on salivary samples of Saharawi celiac children under African-style gluten-free diet (T0), and after 30 (T30) and 60 (T60) days of intervention with Italian-style gluten-free diet.

**Phylum/Family**	**Genus**	**Specie**	**Avg (%) T0**	**Avg (%) T30**	**Avg (%) T60**	**P value**		
						**T0 *vs* T30**	**T0 *vs* T60**	**T30 *vs* T60**
Firmicutes/Aerococcaceae	*Abiotrophia*	*A. defectiva*	0.290	0.375	0.538	0.553	0.075	0.324
Firmicutes/Carnobacteriaceae	*Granulicatella*	*G. adiacens*	0.035	0.135	0.070	0.002	0.119	0.056
		Granulicatella sp	3.038	8.599	6.720	0.000	0.007	0.264
		Gemella sp	3.033	1.835	1.479	0.046	0.001	0.638
Firmicutes/Streptococcaceae	*Streptococcus*	*S. anginosus*	0.052	0.020	0.004	0.160	0.002	0.363
		S. sanguinis	0.545	1.038	0.622	0.128	0.648	0.169
		S. thermophylus	0.111	0.119	0.056	0.865	0.041	0.164
		Streptococcus sp	24.007	20.914	15.413	0.746	0.028	0.079
Firmicutes/Clostridiales Family XI	*Parvinomonas*	*P. micra*	0.217	0.011	0.369	0.002	0.522	0.112

Supplementary Table 4 continued

**Phylum/Family**	**Genus**	**Specie**	**Avg (%) T0**	**Avg (%) T30**	**Avg (%) T60**	**P value**		
						**T0 *vs* T30**	**T0 *vs* T60**	**T30 *vs* T60**
Firmicutes/Clostridiales Family XI	*Eubacterium*	*Eubacterium* sp	0.454	0.064	0.108	0.007	0.017	0.289
Firmicutes/Aerococcaceae	*Mogibacterium*	*Mogibacterium* sp	0.451	0.157	0.117	0.001	0.000	0.352
Firmicutes/Lachnospiraceae	*Catonella*	*Catonella* sp	0.129	0.054	0.034	0.023	0.001	0.391
	Clostridium	*Clostridium* sp	0.183	0.013	0.105	0.009	0.016	0.008
	Moryella	*Moryella* sp	0.009	0.000	0.002	0.035	0.099	0.327
	Oribacterium	*Oribacterium* sp	0.342	0.164	0.191	0.226	0.067	0.482
Firmicutes/Peptococcaceae	*Peptococcus*	*Peptococcus* sp	0.084	0.001	0.012	0.000	0.002	0.083
Firmicutes/Peptostreptococcaceae	*Filifactor*	*Filifactor* sp	0.061[a]	0.003[c]	0.023[b]	0.001	0.036	0.044
	Peptostreptococcus	*Peptostreptococcus* sp	0.402	0.164	0.109	0.026	0.004	0.338
	Selenomonas	*Selenomonas* sp	0.141	0.020	0.090	0.002	0.263	0.015
	Veillonella	*Veillonella* sp	3.779	2.252	1.287	0.107	0.000	0.296
Bacteroidetes/ Poprhyromonadaceae	*Porphyromonas*	*P. endodontalis*	0.151	0.102	0.179	0.544	0.828	0.565

Supplementary Table 4 continued

Phylum/Family	Genus	Specie	Avg (%) T0	Avg (%) T30	Avg (%) T60	P value		
						T0 *vs* T30	T0 *vs* T60	T30 *vs* T60
Bacteroidetes/	*Porphyromonas*	*Porphyromonas* sp	6.452	20.859	14.470	0.001	0.003	0.115
Poprhyromonadaceae	*Tannerella*	*Tannerella* sp	0.046	0.043	0.080	0.871	0.220	0.153
	Prevotella	*P. melaninogenica*	0.156	0.273	0.288	0.043	0.078	0.850
		P. nanceiensis	0.141	0.060	0.101	0.044	0.347	0.248
		P. oris	0.006	0.000	0.010	0.037	0.738	0.327
		P. pallens	2.853	1.649	1.213	0.119	0.015	0.422
		P. tannerae	0.428	0.048	0.093	0.052	0.085	0.418
		P. veroralis	0.043	0.007	0.011	0.007	0.019	0.588
		Prevotella other	19.575	11.596	15.599	0.030	0.020	0.330
Bacteroidetes/Flavobacteriaceae	*Capnocytophaga*	*C. sputigena*	0.002	0.042	0.048	0.000	0.004	0.696
		Capnocytophaga sp	0.219	0.910	1.303	0.002	0.001	0.255
Proteobacteria/Neisseriaceae	*Neisseria*	*Neisseria* sp	7.259	15.529	21.590	0.017	0.000	0.080
	Simonsiella	*Simonsiella* sp	0.007	0.007	0.002	0.945	0.197	0.410

Supplementary Table 4 continued

**Phylum/Family**	**Genus**	**Specie**	**Avg (%) T0**	**Avg (%) T30**	**Avg (%) T60**	**P value**		
						**T0 *vs* T30**	**T0 *vs* T60**	**T30 *vs* T60**
Proteobacteria/ Campylobacteriacaee	*Campylobacter*	*Campylobacter* other	0.213	0.045	0.095	0.001	0.037	0.160
Proteobacteria/Pasteurellaceae	*Aggregatibacter*	*Aggregatibacter segnis*	0.061	0.001	0.003	0.006	0.008	0.542
	Haemophilus	*H. parainfluenzae*	0.892	0.427	0.839	0.226	0.902	0.250
		Haemophilus sp	0.099	0.018	0.026	0.016	0.059	0.768
Fusobacteria/Fusobacteriaceae	*Fusobacterium*	*Fusobacterium* sp	2.992	0.923	3.900	0.001	0.207	0.000
	Leptotrichia	*Leptotrichia* sp	4.409	0.496	3.086	0.000	0.238	0.000
	Streptobacillus	*Streptobacillus* sp	0.007	0.009	0.015	0.853	0.309	0.567
Actinobacteria/Actinomycetaceae	*Actinomyces*	*A. odontolyticus*	0.149	0.092	0.074	0.087	0.034	0.564
		Actinomyces sp	6.736	2.753	2.447	0.000	0.000	0.612
Actinobacteria/Coriobacteriaceae	*Atopobium*	*Atopobium* sp	0.564	0.288	0.196	0.007	0.000	0.352
Actinobacteria/ Corynebacteriaceae	*Corynebacterium*	*C. durum*	0.000	0.022	0.010	0.009	0.105	0.199
		C. matruchotii	0.050	0.005	0.012	0.002	0.013	0.222
		Corynebacterium sp	0.297	0.329	0.338	0.494	0.441	0.259

Supplementary Table 4 continued

Phylum/Family	Genus	Specie	Avg (%) T0	Avg (%) T30	Avg (%) T60	P value		
						T0 *vs* T30	T0 *vs* T60	T30 *vs* T60
Actinobacteria/Micrococcaceae	*Rothia*	*Rothia* sp	0.572	0.622	0.164	0.773	0.006	0.009
Tenericutes/Erysipelotrichaceae	*Bulleidia*	*Solobacterium moorei*	0.178	0.116	0.079	0.219	0.022	0.319
		Bulleidia sp	0.845	0.455	0.414	0.087	0.066	0.813

Data are the means of three independent experiments (n = 3) for each subject. Statistics: t-test; the alpha level: two-tailed.

Supplementary Table 5. Concentration (ppm) of volatile organic compounds, which were found on salivary samples of Saharawi celiac children under African-style gluten-free diet (T0), and after 30 (T30) and 60 (T60) days of intervention with Italian-style gluten-free diet.

**Chemical class**	**T0**		**T30**		**T60**		**P value**		
	Median	Range	Median	Range	Median	Range	**T0 *vs* T30**	**T0 *vs* T60**	**T30 *vs* T60**
Alcohols									
1-Butanol	0.03	0.00-0.07	0.00	0.00-0.03	0.00	0.00-0.03	0.000	0.000	0.734
1-Hexanol	0.01	0.00-0.34	0.00	0.00-0.00	0.00	0.00-0.00	0.134	0.134	0.112
2-Hexanol	0.03	0.00-0.16	0.08	0.00-0.32	0.05	0.00-0.25	0.108	0.189	0.592
2-Methyl-1-propanol	0.00	0.00-0.06	0.03	0.00-0.19	0.03	0.00-0.43	0.029	0.051	0.665
3-Methyl-3-buten-1-ol	0.00	0.00-0.05	0.84	0.03-4.45	0.83	0.10-4.05	0.001	0.000	0.838
4-Methoxybenzhydrol	0.03	0.00-0.08	1.46	0.16-5.11	1.23	0.16-5.56	0.000	0.001	0.439
Ethanol	4.23	0.39-5.34	5.50	2.38-14.76	4.47	1.07-7.85	0.019	0.291	0.049
Phenol, 2,4-bis(1,1-dimethylethyl)	0.09	0.02-0.33	0.36	0.06-1.77	0.26	0.07-1.88	0.006	0.012	0.975
Phenol, 2,5-bis(1,1-dimethylethyl)	0.20	0.06-1.52	0.00	0.00-0.09	0.00	0.00-0.10	0.001	0.001	0.516
Phenol, 4-(1,1,3,3-tertamethylbutyl)-	3.39	0.79-9.09	0.00	0.00-0.15	0.00	0.00-0.020	0.000	0.000	0.636
Phenol, 4-(1,1-dimethylpropyl)-	0.49	0.08-2.40	0.00	0.00-0.10	0.00	0.00-0.11	0.000	0.000	0.908
Phenyl alcohol (phenol)	0.34	0.10-0.83	0.04	0.00-0.17	0.02	0.00-0.34	0.117	0.117	0.993
Propyl alcohol (1-propanol)	3.37	0.06-6.41	0.12	0.00-0.32	0.05	0.00-0.24	0.000	0.000	0.026

Supplementary Table 5 continued

Chemical class	T0		T30		T60		P value		
	Median	Range	Median	Range	Median	Range	**T0 *vs* T30**	**T0 *vs* T60**	**T30 *vs* T60**
Total alcohols	14.42	7.63-22.06	9.39	4.57-22.34	7.72	4.81-14.09	0.11	0.00	0.08
Aldehydes									
2-Octenal (E)	0.00	0.00-0.23	0.00	0.00-0.07	0.00	0.00-0.02	0.520	0.167	0.051
Decanal	0.02	0.00-0.08	0.01	0.00-0.52	0.02	0.00-0.79	0.250	0.250	0.753
Hexanal	0.01	0.00-1.00	0.00	0.00-0.02	0.00	0.00-0.02	0.204	0.198	0.542
Nonanal	4.71	1.03-37.21	0.00	0.00-0.57	0.01	0.00-0.20	0.003	0.003	0.965
Octanal	1.01	0.38-3.45	0.00	0.00-0.05	0.00	0.00-0.18	0.000	0.000	0.139
Total aldehydes	5.38	1.97-40.8	0.10	0.00-1.11	0.14	0.00-1.54	0.001	0.001	0.811
Ethers									
1,1-Oxybis-heptane	0.00	0.00-0.01	2.29	0.10-10.86	0.93	0.20-10.02	0.004	0.007	0.629
Total ethers	0.00	0.00-0.01	2.29	0.10-10.86	0.93	0.20-10.02	0.004	0.007	0.629
Esters									
Butanoic acid, 2-methyl-, octyl ester	0.04	0.00-0.09	0.00	0.00-0.08	0.01	0.00-0.26	0.068	0.782	0.153
Dichloroacetic acid, 2-octyl ester	0.00	0.00-0.00	0.01	0.00-0.11	0.00	0.00-0.11	0.023	0.015	0.511
Ethyl acetate	0.04	0.01-0.15	0.44	0.17-0.83	0.52	0.13-1.42	0.000	0.000	0.159
Heptanoic acid, ethyl ester	0.01	0.00-0.02	0.00	0.00-0.02	0.00	0.00-0.03	0.000	0.015	0.428

Supplementary Table 5 continued

Chemical class	T0		T30		T60		P value		
	Median	Range	Median	Range	Median	Range	**T0 *vs* T30**	**T0 *vs* T60**	**T30 *vs* T60**
Octanoic acid, ethyl ester	0.00	0.00-0.13	0.00	0.00-0.00	0.00	0.00-0.05	0.072	0.144	0.325
Total esters	0.09	0.04-0.25	0.51	0.17-0.91	0.55	0.25-1.46	0.000	0.000	0.095
Aromatic heterocyclic									
1(3H)-Isobenzofuranone (γ-lactone)	0.09	0.00-0.17	1.70	0.48-5.19	1.70	0.30-6.19	0.000	0.000	0.627
(R)-5,6-Dihydro-6-pentyl-2H-Pyran-2-one	0.00	0.00-0.10	2.33	0.28-8.04	2.36	0.32-4.09	0.000	0.000	0.548
2-Pentyl furan	1.02	0.08-3.80	0.02	0.00-0.12	0.02	0.00-0.25	0.000	0.000	0.474
Furan	0.09	0.00-0.24	1.05	0.30-2.14	3.22	0.95-9.03	0.000	0.000	0.001
Total aromatic heterocyclic	1.25	0.29-4.00	5.23	1.47-15.37	7.17	4.53-16.11	0.000	0.000	0.247
Hydrocarbons									
1,2,3-Trimethyl-benzene	0.09	0.02-0.87	0.04	0.00-0.34	0.02	0.00-1.05	0.103	0.428	0.544
1,3-Bis(1,1-dimethylethyl)-benzene	5.94	0.76-25.20	1.34	0.39-4.84	1.48	0.53-18.57	0.002	0.046	0.249
1-Chloro decane	0.53	0.06-7.72	0.01	0.00-0.06	0.00	0.00-0.10	0.023	0.024	0.336
1-Methyl-2-(1-methylethyl)-benzene	0.02	0.00-0.72	0.00	0.00-0.05	0.00	0.00-0.01	0.063	0.042	0.038
1-Octadecene	2.28	0.16-3.86	0.00	0.00-0.41	0.00	0.00-0.37	0.000	0.000	0.842
1-Undecene	0.00	0.00-0.00	0.03	0.00-0.13	0.03	0.00-0.21	0.002	0.005	0.588
2,4-Dimethyl-1-heptene	0.03	0.00-0.04	2.29	0.38-3.70	2.51	0.75-16.21	0.000	0.001	0.105
3-Carene	0.00	0.00-0.03	0.70	0.00-3.91	0.82	0.00-2.80	0.005	0.002	0.869

Supplementary Table 5 continued

Chemical class	T0		T30		T60		P value		
	Median	Range	Median	Range	Median	Range	T0 *vs* T30	T0 *vs* T60	T30 *vs* T60
4-Methyl-1-pentene	0.00	0.00-0.36	0.03	0.00-0.45	0.03	0.00-0.43	0.249	0.425	0.665
Benzene	0.14	0.00-0.39	0.01	0.00-0.04	0.01	0.00-0.05	0.000	0.000	0.339
Benzene (octyloxy)	0.04	0.00-0.09	0.01	0.00-0.03	0.00	0.00-0.04	0.001	0.000	0.309
Ethylbenzene	0.01	0.00-0.10	0.01	0.00-0.04	0.02	0.00-0.09	0.468	0.649	0.160
Hexadecane	0.02	0.00-0.06	0.00	0.00-0.05	0.00	0.00-0.05	0.134	0.079	0.810
Nonadecane	0.01	0.00-0.04	0.07	0.00-0.33	0.00	0.00-0.07	0.024	0.449	0.008
Pentadecane	0.04	0.00-0.12	0.00	0.00-0.17	0.04	0.00-0.25	0.786	0.773	0.992
Toluene	0.36	0.00-0.80	1.47	0.34-3.55	1.75	0.34-3.90	0.000	0.000	0.767
Trichloromethane	0.25	0.00-0.97	0.00	0.00-0.40	0.00	0.00-0.04	0.001	0.000	0.373
Limonene	0.00	0.00-0.22	2.02	0.00-8.56	0.42	0.00-1.97	0.001	0.003	0.012
Heptylcyclohexane	0.03	0.01-0.07	0.08	0.00-0.18	0.04	0.01-0.13	0.023	0.152	0.333
Total hydrocarbons	12.26	6.40-19.84	9.44	1.52-15.82	8.19	3.24-42.00	0.115	0.577	0.608
Ketones									
2,6-Dimethyl-4-heptanone	0.00	0.00-0.03	0.00	0.00-1.76	0.00	0.00-1.54	0.177	0.344	0.635
2-Butanone	0.02	0.00-0.11	6.79	2.41-19.04	7.31	2.53-24.27	0.000	0.000	0.729
2-Methyl-3-decen-5-one	0.01	0.00-0.04	0.12	0.00-0.39	0.08	0.00-0.67	0.00	0.027	0.939
2-Nonanone	0.00	0.00-0.03	0.00	0.00-1.76	0.02	0.00-1.54	0.032	0.075	0.423
3-(but-3-enyl)-Cyclohexanone	0.02	0.00-0.008	0.00	0.00-0.20	0.00	0.00-0.12	0.404	0.085	0.096

Supplementary Table 5 continued

Chemical class	T0		T30		T60		P value		
	Median	Range	Median	Range	Median	Range	**T0 *vs* T30**	**T0 *vs* T60**	**T30 *vs* T60**
3-Methyl-2-butanone	0.08	0.00-0.26	3.47	1.20-5.84	4.20	1.95-16.93	0.000	0.000	0.086
4-Methyl-2-hexanone	0.00	0.00-0.20	0.02	0.00-0.12	0.00	0.00-0.14	0.723	0.822	0.413
4-Methyl-3-penten-2-one	0.57	0.36-2.14	1.70	0.61-3.43	1.81	0.56-3.48	0.003	0.003	0.960
Acetone	0.00	0.00-0.16	4.15	2.21-9.69	4.02	1.83-11.12	0.000	0.000	0.775
Methyl isobutyl ketone/2-hexanone	0.10	0.00-0.13	0.02	0.01-0.04	0.02	0.02-0.03	0.000	0.000	0.871
Total ketones	1.00	0.57-2.49	18.55	6.72-25.85	17.40	10.32-50.04	0.000	0.000	0.373
Terpenes									
Copaene	0.00	0.00-0.01	0.84	0.00-2.24	0.62	0.00-2.28	0.000	0.001	0.656
Caryophyllene	0.01	0.00-0.04	1.04	0.26-3,45	0.98	0.21-4.18	0.000	0.000	0.478
Menthol	0.02	0.01-4.68	0.00	0.00-0.00	0.00	0.00-0.00	0.239	0.231	0.325
Total terpenes	0.04	0.00-4.70	2.05	0.70-4.95	1.58	0.28-4.93	0.003	0.012	0.486
Thiophenes									
2-(3-methylbutyl)-Thiophene	0.03	0.01-0.05	4.13	1.21-10.38	3.04	0.58-19.52	0.000	0.005	0.445
2-Pentyl-thiophene	11.06	1. 92-29.41	26.96	11.65-72.81	22.13	9,46-43.81	0.000	0.016	0.450
Total tiofenes	11.09	1.94-29.46	29.01	14.48-74.02	26.30	10.65-119.27	0.000	0.011	0.427
Organic acids									
2,2-Dimethyl-propanoic acid	0.00	0.00-0.002	0.02	0.00-0.36	0.03	0.00-0.31	0.027	0.009	0.907
Total organic acids	0.00	0.00-0.002	0.02	0.00-0.36	0.03	0.00-0.31	0.027	0.009	0.907

Supplementary Table 5 continued

**Chemical class**	**T0**		**T30**		**T60**		**P value**		
	Median	Range	Median	Range	Median	Range	**T0 *vs* T30**	**T0 *vs* T60**	**T30 *vs* T60**
n.d.2	0.00	0.00-0.00	0.00	0.00-0.09	0.00	0.00-0.09	0.112	0.292	0.615
n.d.3 (ion 97)	2.29	0.28-5,38	0.24	0.00-1.19	0.16	0.04-1.05	0.000	0.000	0.262
n.d.4 (ion 102)	2.45	0.00-5.66	0.25	0.00-1.81	0.21	0.00-0.91	0.000	0.000	0.218
Total ions	4.74	0.33-11.04	0.51	0.01-2.89	0.39	0.04-2.05	0.000	0.000	0.208

Data are the means of three independent experiments (n = 3) for each subject. Statistics: t-test; the alpha level: two-tailed.

Figure S1. Averaged number of species (OTU), richness (Chao 1) and diversity (Shannon index) of salivary samples of Saharawi celiac children under African-style gluten-free diet (T0), and after 30 (T30) and 60 (T60) days of intervention with Italian-style gluten-free diet. The centred line of each box represents the median ( ), the top and bottom of the box represent the 75th and 25th percentile of the data, respectively. The top and bottom of the error bars represent the 5th and 95th percentile of the data, respectively. The circles in each box plot extend to the outliers of the data.

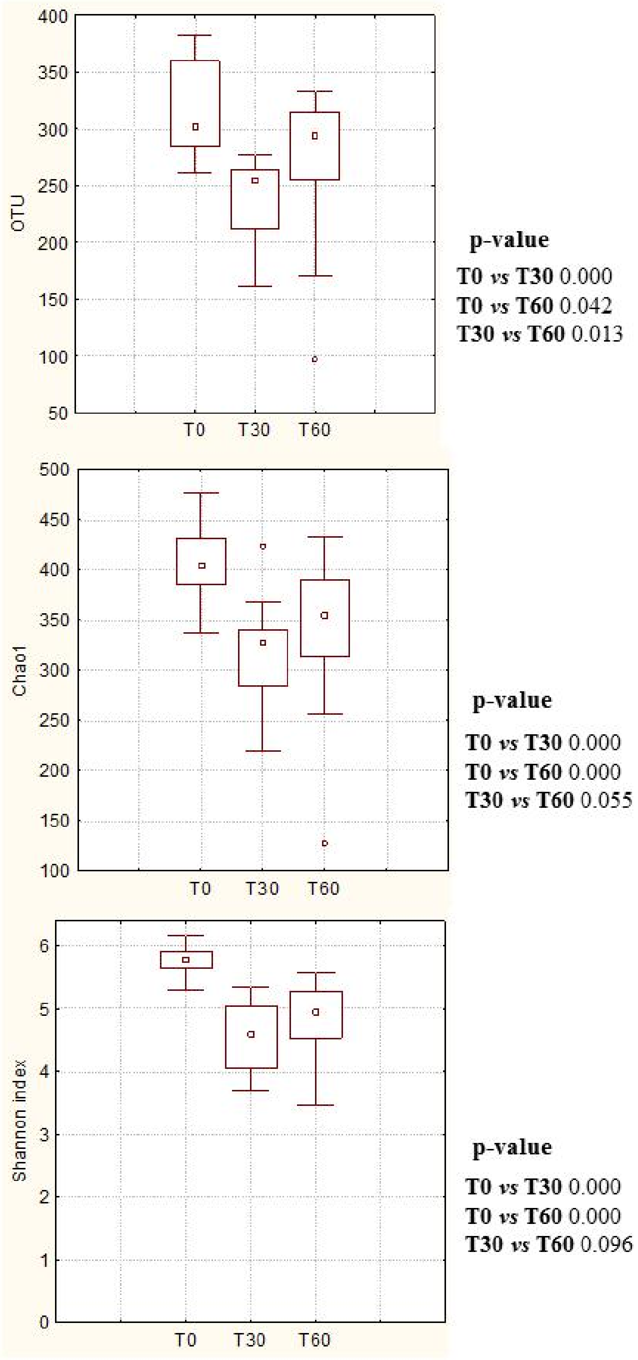

Figure S2. Relative abundance (%) of total bacteria found at the phylum level on salivary samples of Saharawi celiac children under African-style gluten-free diet (T0), and after 30 (T30) and 60 (T60) days of intervention with Italian-style gluten-free diet.

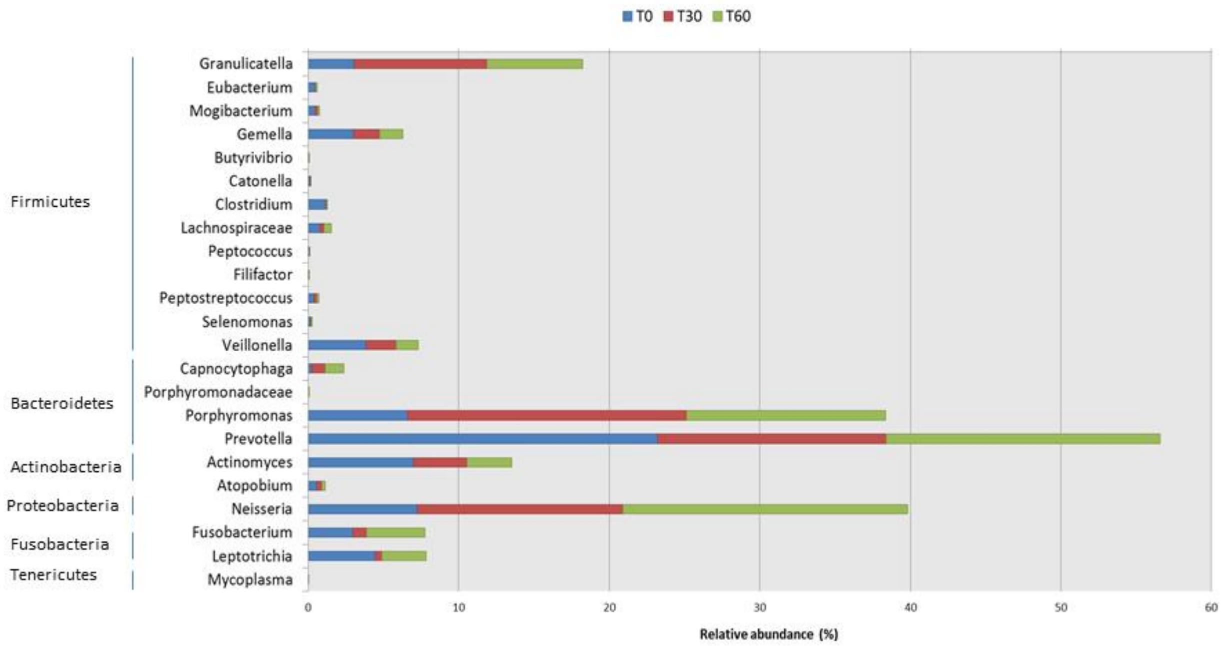

Figure S3. Relative proportions (%) of predominant bacteria at family level, which showed significant (P<0.050) differences between the salivary samples of Saharawi celiac children under African-style gluten-free diet (T0), and after 30 (T30) and 60 (T60) days of intervention with Italian-style gluten-free diet.

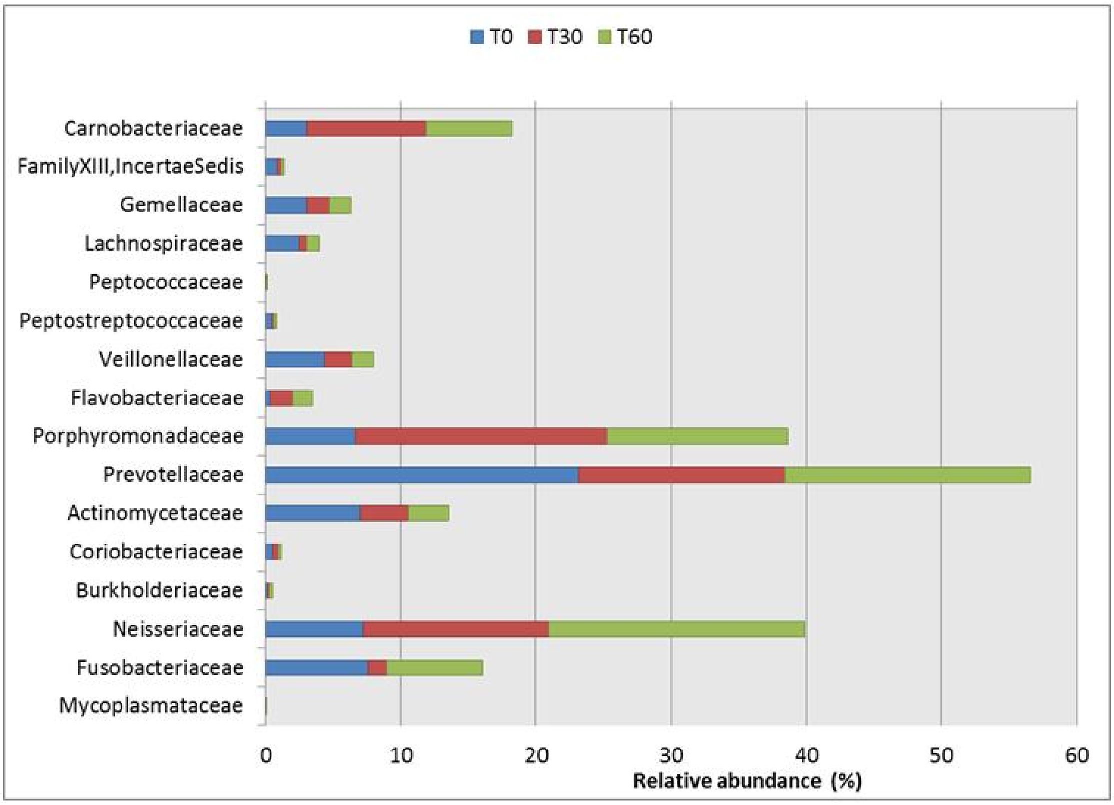

Figure S4. Significant co-occurrence and co-exclusion relationships between bacteria at family level. Spearman's rank correlation matrix was used. Strong correlations are indicated by large circles, whereas weak correlations are indicated by small circles. The colors of the scale bar denote the nature of the correlation, with 1 indicating a perfectly positive correlation (dark blue) and -1 indicating a perfectly negative correlation (dark red) between two microbial genera (families). Only significant correlations (FDR<0.050) are shown. Bacterial phyla are shown on the left side.

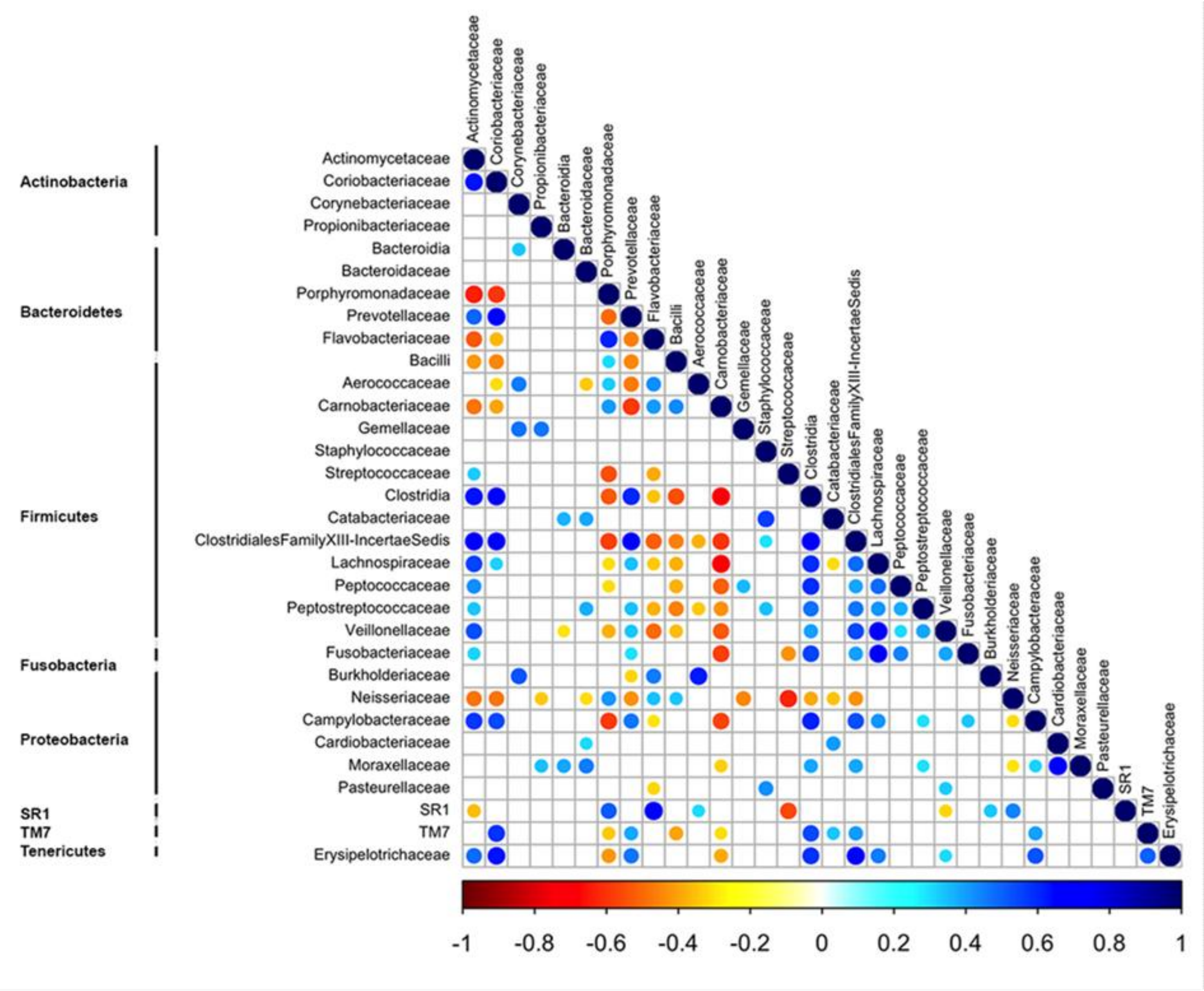

Figure S5. Significant co-occurrence and co-exclusion relationships between bacteria at genus level. Spearman's rank correlation matrix was used. Strong correlations are indicated by large circles, whereas weak correlations are indicated by small circles. The colors of the scale bar denote the nature of the correlation, with 1 indicating a perfectly positive correlation (dark blue) and -1 indicating a perfectly negative correlation (dark red) between two microbial genera (families). Only significant correlations (FDR<0.05) are shown. Bacterial phyla are shown on the left side.

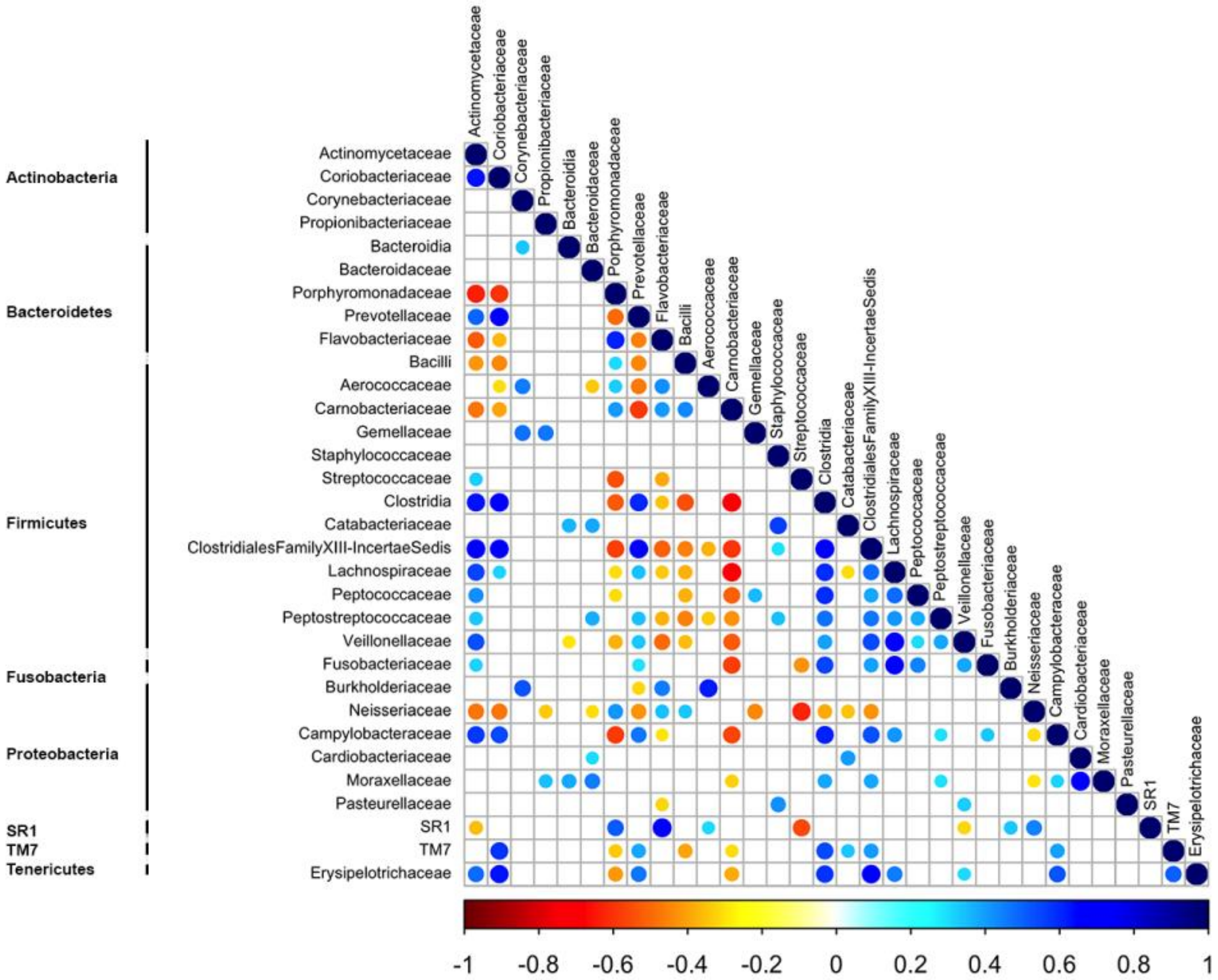

Figure S6. Principle Coordinate Analysis based on unweighted UniFrac analysis of all 16S rRNA gene sequences, which were found on salivary samples of Saharawi celiac children under African-style gluten-free diet (T0), and after 30 (T30) and 60 (T60) days of intervention with Italian-style gluten-free diet, and of Italian celiac children, under gluten-free diet for at least two years (T-CD), and healthy children (HC).

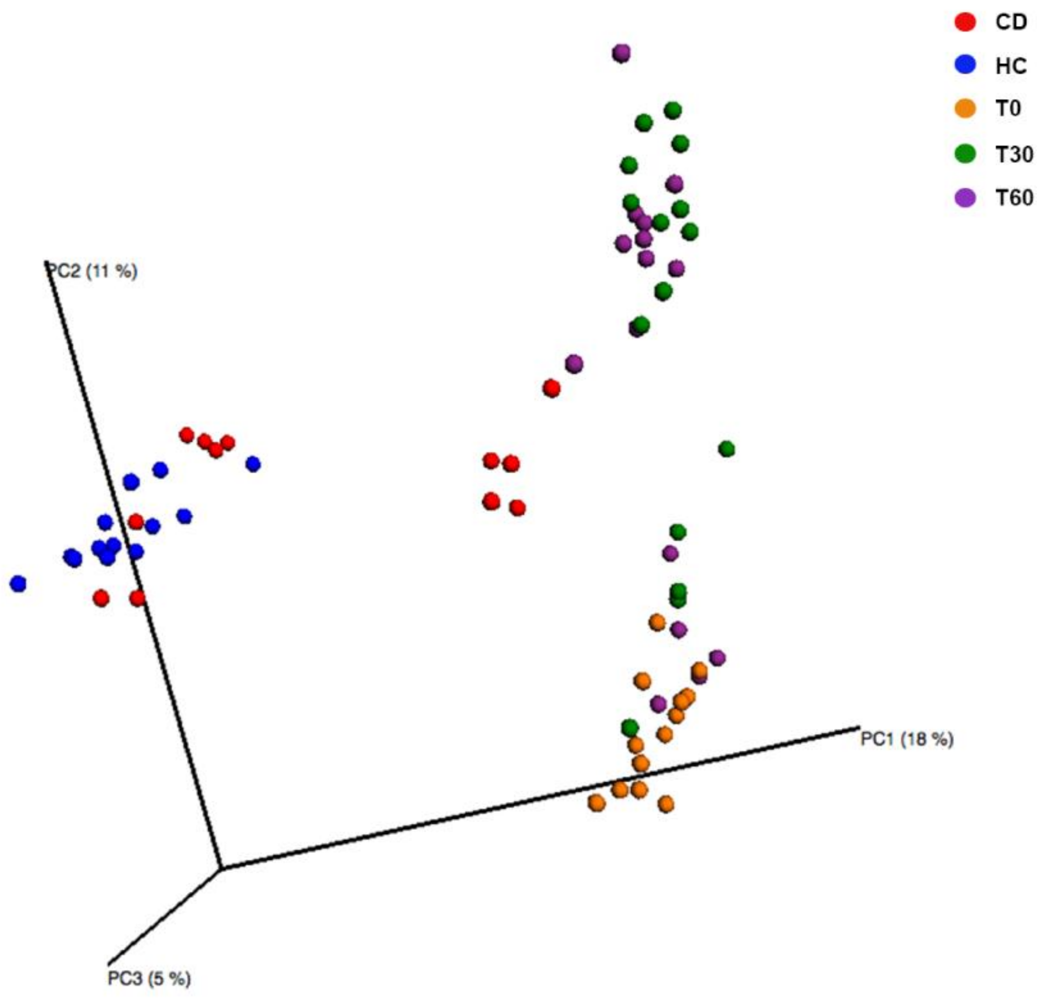

Figure S7. Procrustes analysis combining Bray Curtis Principle Coordinate Analysis of microbiota and predicted metagenome for the saliva samples analyzed in this study.

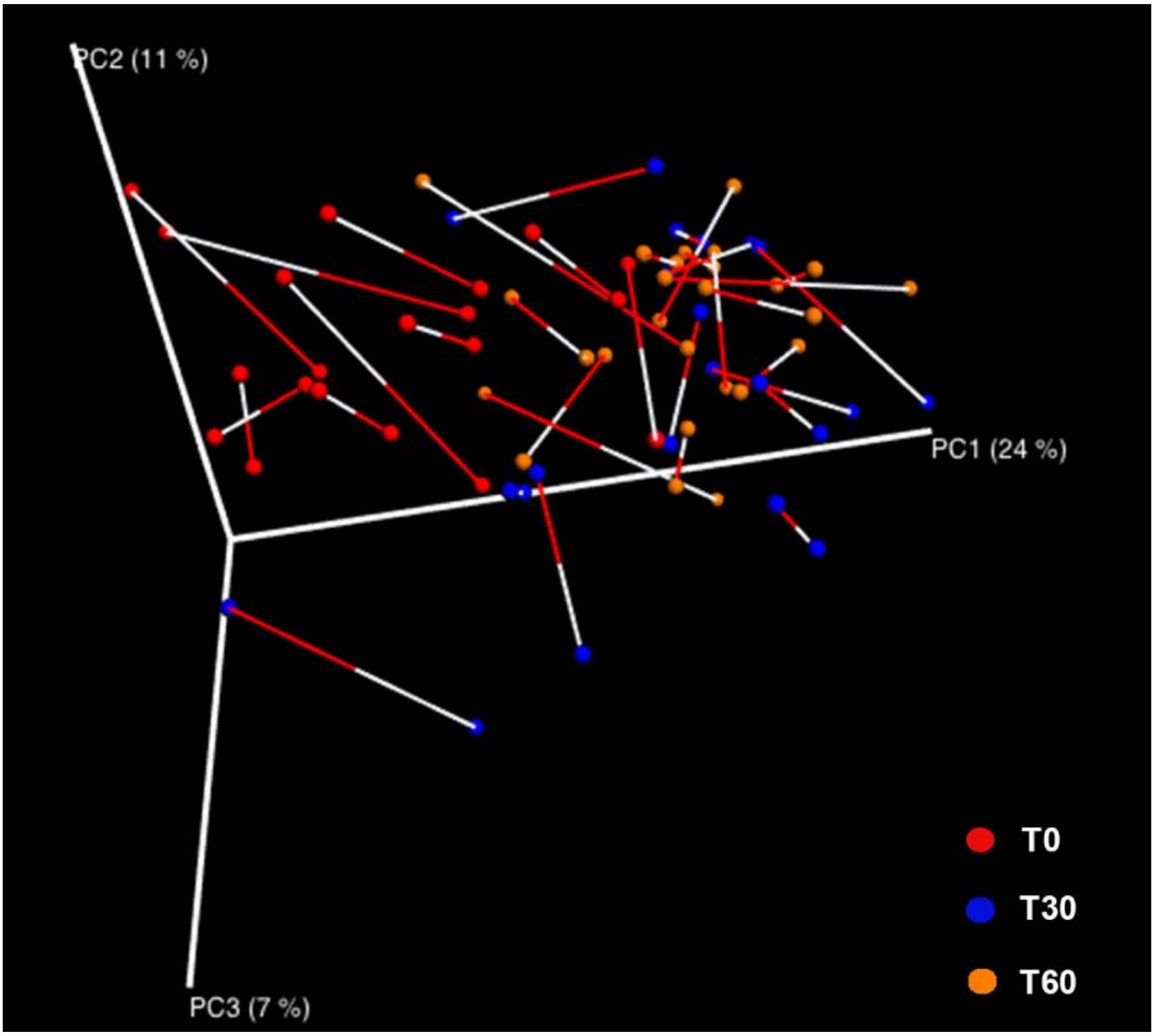

Figure S8. Total concentration (ppm) of volatile organic compounds, which were found in the salivary samples of Saharawi celiac children under African-style gluten-free diet (T0), and after 30 (T30) and 60 (T60) days of intervention with Italian-style gluten-free diet. Treated celiac children (T-CD) were numbered.

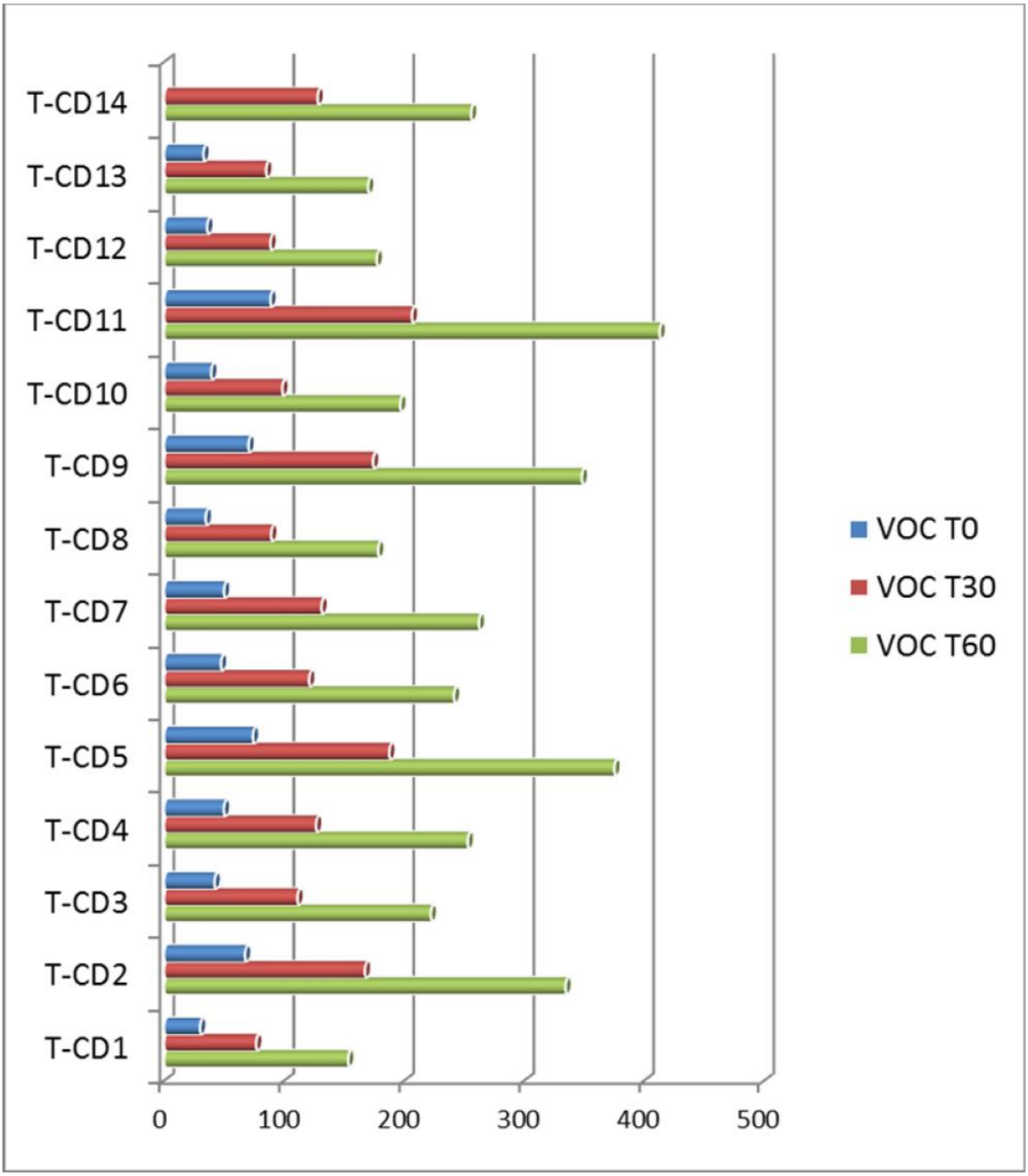

Figure S9. Spearman's rank correlation matrix of significant (FDR<0.05) relationships between bacteria (at genus level) and volatile organic compounds, calculated for saliva samples after 0 (T0), 30 (T30) and 60 (T60) days of treatment. Strong correlations are indicated by large circles, whereas weak correlations are indicated by small circles. The colors of the scale bar denote the nature of the correlation, with 1 indicating a perfectly positive correlation (dark blue) and -1 indicating a perfectly negative correlation (dark red) between microbial genera and metabolites. Only significant correlations (FDR<0.050) are shown.

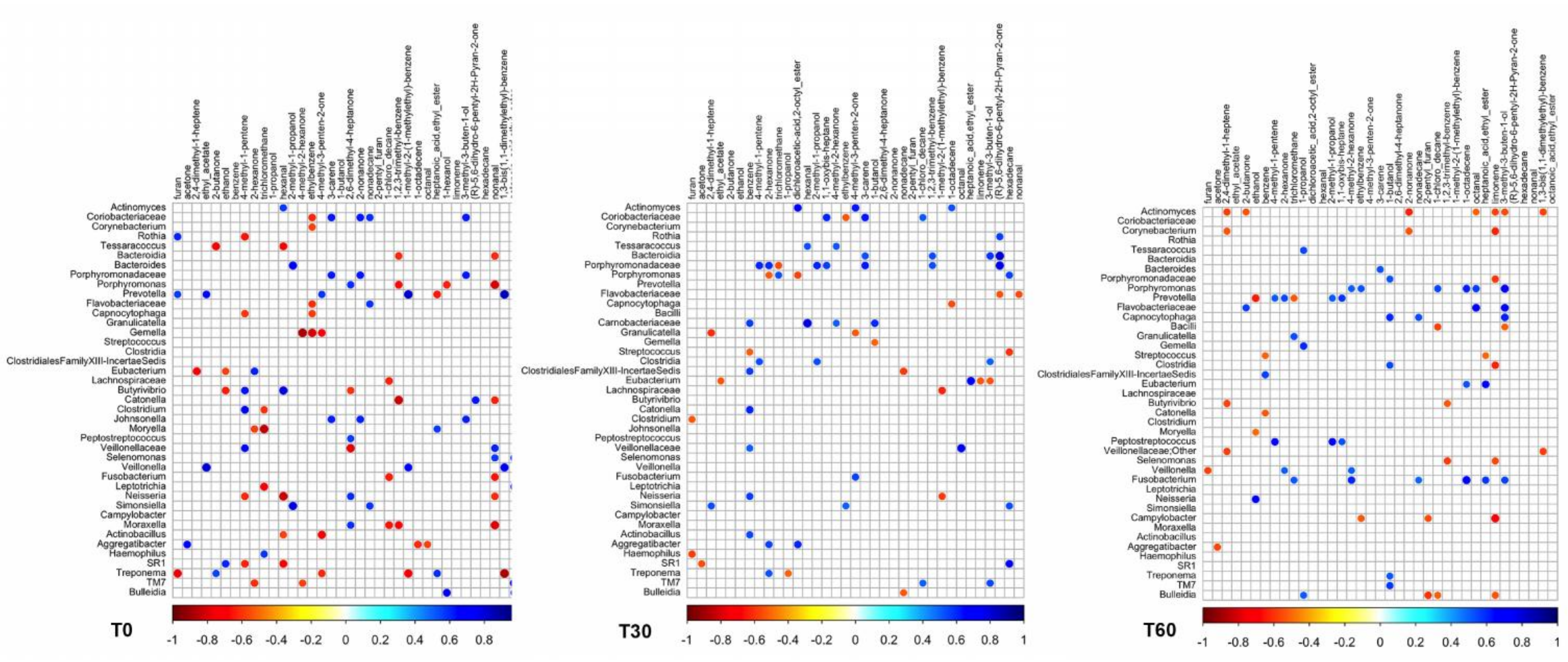

Figure S10. Significant correlations between volatile organic compounds (filtered by a subject prevalence of 5%) and dietary information. The colors of the scale bar denote the nature of the correlation, with 1 indicating a perfectly positive correlation (read) and -1 indicating a perfectly negative correlation (green). Only significant correlations (FDR<0.05) are shown.

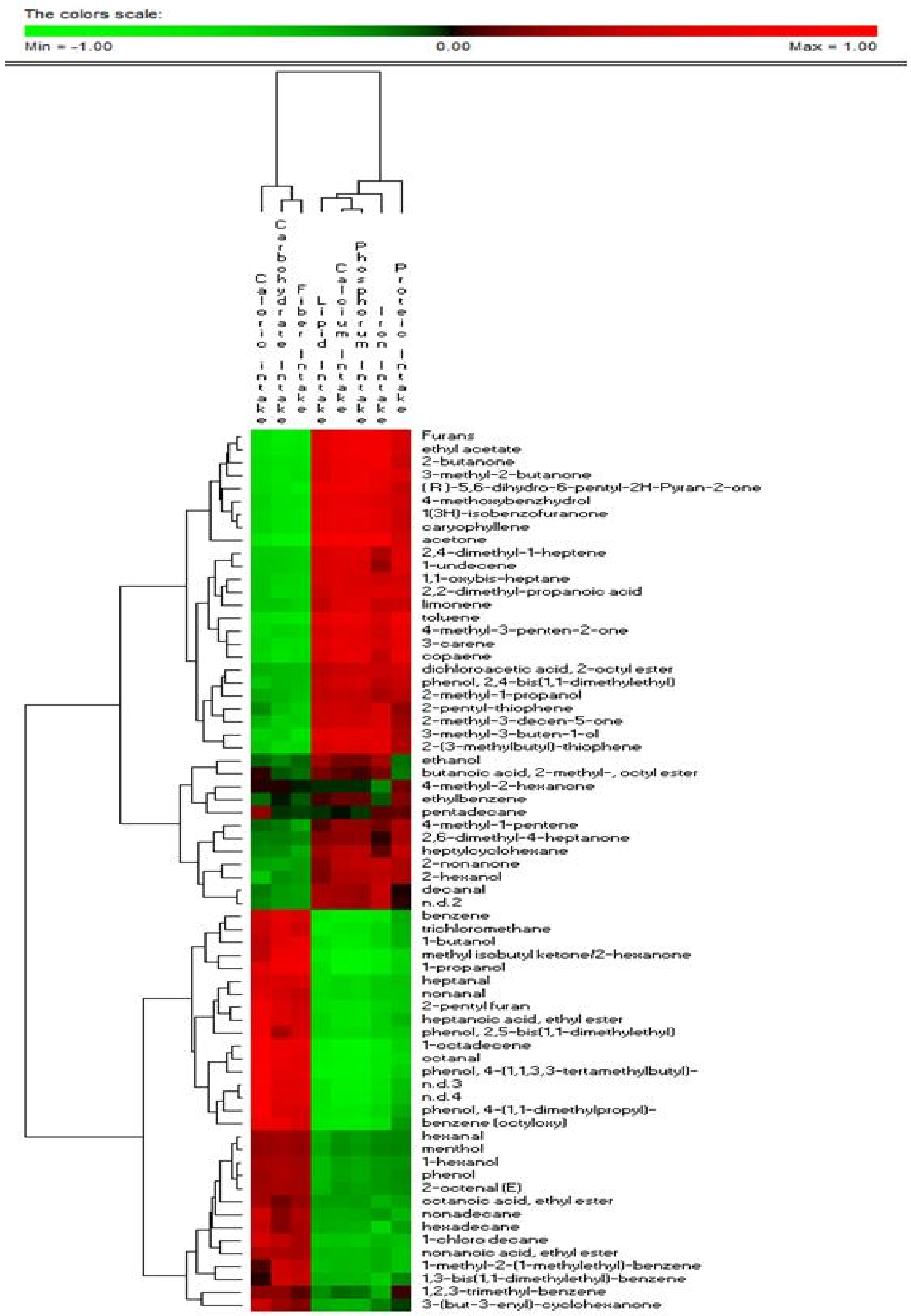

Supplement: Supplementary Information [file srep18571-s1.pdf]
